# Integrative analysis of genomic alterations in triple-negative breast cancer in association with homologous recombination deficiency

**DOI:** 10.1371/journal.pgen.1006853

**Published:** 2017-06-21

**Authors:** Masahito Kawazu, Shinya Kojima, Toshihide Ueno, Yasushi Totoki, Hiromi Nakamura, Akiko Kunita, Wei Qu, Jun Yoshimura, Manabu Soda, Takahiko Yasuda, Natsuko Hama, Mihoko Saito-Adachi, Kazuhito Sato, Shinji Kohsaka, Eirin Sai, Masako Ikemura, Shigeru Yamamoto, Tomoko Ogawa, Masashi Fukayama, Keiichiro Tada, Yasuyuki Seto, Shinichi Morishita, Shoichi Hazama, Tatsuhiro Shibata, Yoshihiro Yamashita, Hiroyuki Mano

**Affiliations:** 1 Department of Medical Genomics, Graduate School of Medicine, The University of Tokyo, Tokyo, Japan; 2 Department of Cellular Signaling, Graduate School of Medicine, The University of Tokyo, Tokyo, Japan; 3 Division of Cancer Genomics, National Cancer Center Research Institute, Tokyo, Japan; 4 Department of Pathology, Graduate School of Medicine, The University of Tokyo, Tokyo, Japan; 5 Department of Computational Biology and Medical Sciences, Graduate School of Frontier Sciences, The University of Tokyo, Chiba, Japan; 6 Department of Gastroenterological, Breast and Endocrine Surgery, Yamaguchi University Graduate School of Medicine, Yamaguchi, Japan; 7 Department of Breast Surgery, Mie University Hospital, Mie, Japan; 8 Department of Breast and Endocrine Surgery, Graduate School of Medicine, The University of Tokyo, Tokyo, Japan; 9 Laboratory of Molecular Medicine, Human Genome Center, Institute of Medical Science, The University of Tokyo, Tokyo, Japan; 10 National Cancer Center Research Institute, Tokyo, Japan; Dana Farber Cancer Institute, UNITED STATES

## Abstract

Triple-negative breast cancer (TNBC) cells do not express estrogen receptors, progesterone receptors, or human epidermal growth factor receptor 2. Currently, apart from poly ADP-ribose polymerase inhibitors, there are few effective therapeutic options for this type of cancer. Here, we present comprehensive characterization of the genetic alterations in TNBC performed by high coverage whole genome sequencing together with transcriptome and whole exome sequencing. Silencing of the *BRCA1* gene impaired the homologous recombination pathway in a subset of TNBCs, which exhibited similar phenotypes to tumors with *BRCA1* mutations; they harbored many structural variations (SVs) with relative enrichment for tandem duplication. Clonal analysis suggested that *TP53* mutations and methylation of CpG dinucleotides in the *BRCA1* promoter were early events of carcinogenesis. SVs were associated with driver oncogenic events such as amplification of *MYC*, *NOTCH2*, or *NOTCH3* and affected tumor suppressor genes including *RB1*, *PTEN*, and *KMT2C*. Furthermore, we identified putative *TGFA* enhancer regions. Recurrent SVs that affected the *TGFA* enhancer region led to enhanced expression of the *TGFA* oncogene that encodes one of the high affinity ligands for epidermal growth factor receptor. We also identified a variety of oncogenes that could transform 3T3 mouse fibroblasts, suggesting that individual TNBC tumors may undergo a unique driver event that can be targetable. Thus, we revealed several features of TNBC with clinically important implications.

## Author summary

Cancer can result from genetic alterations, some of which can be good drug targets. To reveal genetic alterations that provide important information for the development of ideal therapeutic strategies for triple-negative breast cancer (TNBC), TNBC tumor samples were subjected to comprehensive genomic analyses. We identified novel recurrent structural variations associated with enhanced expression of the *TGFA* gene that encodes one of the high affinity ligands for epidermal growth factor receptor (EGFR). Although *TGFA* expression is known to be elevated in a subset of TNBC tumors, this is the first report of the mechanistic basis of this phenomenon. It is of particular importance considering that anti-EGFR agents are possible therapeutic options for TNBC patients. Our study also revealed several features associated with “BRCAness”, which is critical for identification of patients who may be responsive to platinum agents and/or poly ADP-ribose polymerase inhibitors. Thus, the data presented in this report may advance our understanding of the pathogenesis of TNBC.

## Introduction

Triple-negative breast cancer (TNBC) comprises 15–20% of all breast cancers (BCs) and is defined by a lack of estrogen and progesterone receptor expression and the absence of *ERBB2* gene amplification, which encodes human epidermal growth factor receptor 2 (HER2) [1]. Recent advances in sequencing technology have provided meaningful genomic and epigenomic insights into the pathogenesis of BC types including TNBC [2–5]. Mutations of *TP53* [2,4,5], loss-of-function of *BRCA1* [6–8], and amplification and enhanced expression of *MYC* [9] are common events in TNBC. Because it is difficult to specifically target *MYC*, cytotoxic chemotherapy remains the only approved treatment.

Poly ADP-ribose polymerase (PARP) inhibitors are newly developed treatment options for a subset of TNBCs [10]. Tumors with a defective homologous recombination (HR) pathway are expected to be susceptible to PARP inhibitors, because tumor cells cannot tolerate additional DNA damage in the absence of HR pathway proteins and DNA damage repair mechanisms mediated by PARP. At present, only tumors with mutations in *BRCA1* or *BRCA2* have been shown to be responsive to PARP inhibitors. Thus, identification of biomarkers that distinguish responders to PARP inhibitors is required [11].

Deconvolution of oncogenic events can contribute to the development of targeted therapy for cancer because oncogenes can be ideal therapeutic targets. For example, treatment of BC with *HER2* amplification is greatly improved by the use of an anti-HER2 agent [12]. Identification of *EML4*-*ALK* fusion genes in lung adenocarcinoma has led to the application of ALK inhibitors for the treatment of lung adenocarcinoma with *EML4*-*ALK* fusions [13]. Although alterations have been reported in certain oncogenes, such as those involved in the phosphatidylinositol-3-kinase-AKT pathway [3] or NOTCH pathway [14,15], the frequency of these oncogenic events appears to be relatively low in TNBC. It is likely that many rare oncogenes remain to be identified in TNBC, which constitute the “long tail” [16].

Comprehensive analysis of the TNBC genome has often been hampered by low tumor content in a given specimen because of the presence of stroma and/or necrotic tissue. Thus, we characterized the genomic alterations of TNBC to identify oncogenic gene alterations by high coverage whole genome sequencing (WGS) combined with whole exome sequencing (WES) and transcriptome sequencing (RNA-Seq). To assess the tumorigenic potential of candidate oncogenes with high probability, we also employed biological assays for transformation [17] where possible.

We describe the molecular phenotypes of tumors with a defective HR pathway in detail, providing fundamental information for the development of treatment strategies involving PARP inhibitors. Our observations also support the notion that SVs in TNBC affect tumor suppressor genes and oncogenes, as suggested in previous reports [6,18]. As one of the oncogenes affected by SVs, we have identified *TGFA*, a gene encoding one of the ligands for the epidermal growth factor receptor. Upregulation of *TGFA* expression has been reported in a subset of TNBC [19], and enhanced expression of *TGFA* is associated with BC development [20]. However, the mechanistic basis of enhanced *TGFA* expression has not yet been elucidated. In this study, we present the possible mechanisms of *TGFA* activation. We also identified several rare oncogenes and confirmed their tumorigenic potential in biological assays. Here, we present several important findings that could advance our understanding of TNBC pathogenesis.

## Results

### Mutational signatures define the molecular phenotype of TNBC

To comprehensively characterize the genetic alterations that occur in TNBC, we subjected 36 surgically resected TNBC tissues to WES, together with paired normal tissues. We further analyzed 23 tumors out of these 36 tumors, together with 17 estrogen receptor-positive (ER+) and 15 HER2-positive (HER2+) BC samples, using RNA-seq (S1 Table). WES identified a median of 52 (range, 3–170) somatic nonsynonymous single nucleotide variations (SNVs) and a median of 5 (range, 0–20) somatic insertions/deletions (indels) in the coding regions of TNBC samples. Samples with expected high tumor content (> 30%, 16 samples) were subjected to deep WGS (S1 Table), yielding a median of 162 (range, 137.5–173.9) mean coverage (S2 Table). WGS identified a median of 102.5 (range, 46–193) somatic nonsynonymous SNVs in the exonic regions and a median of 5.1 (range, 2.8–7.9) SNVs per megabase. We also found a median of 6.5 (range, 1–15) somatic indels in the exonic regions and a median of 0.11 (range, 0.048–0.22) indels per megabase in the entire genome (S2 Table). Frequencies of SNVs and indels were in good agreement with previous reports [2–5]. As previously reported [2–5], we observed a high frequency of *TP53* mutations (72%) and relatively low frequency of *PIK3CA* mutations (19%) (S1 Fig). In the majority of tumors with *TP53* mutations, expression of the mutant allele was greater than that of the wild-type allele (S1 Fig).

**Fig 1:**
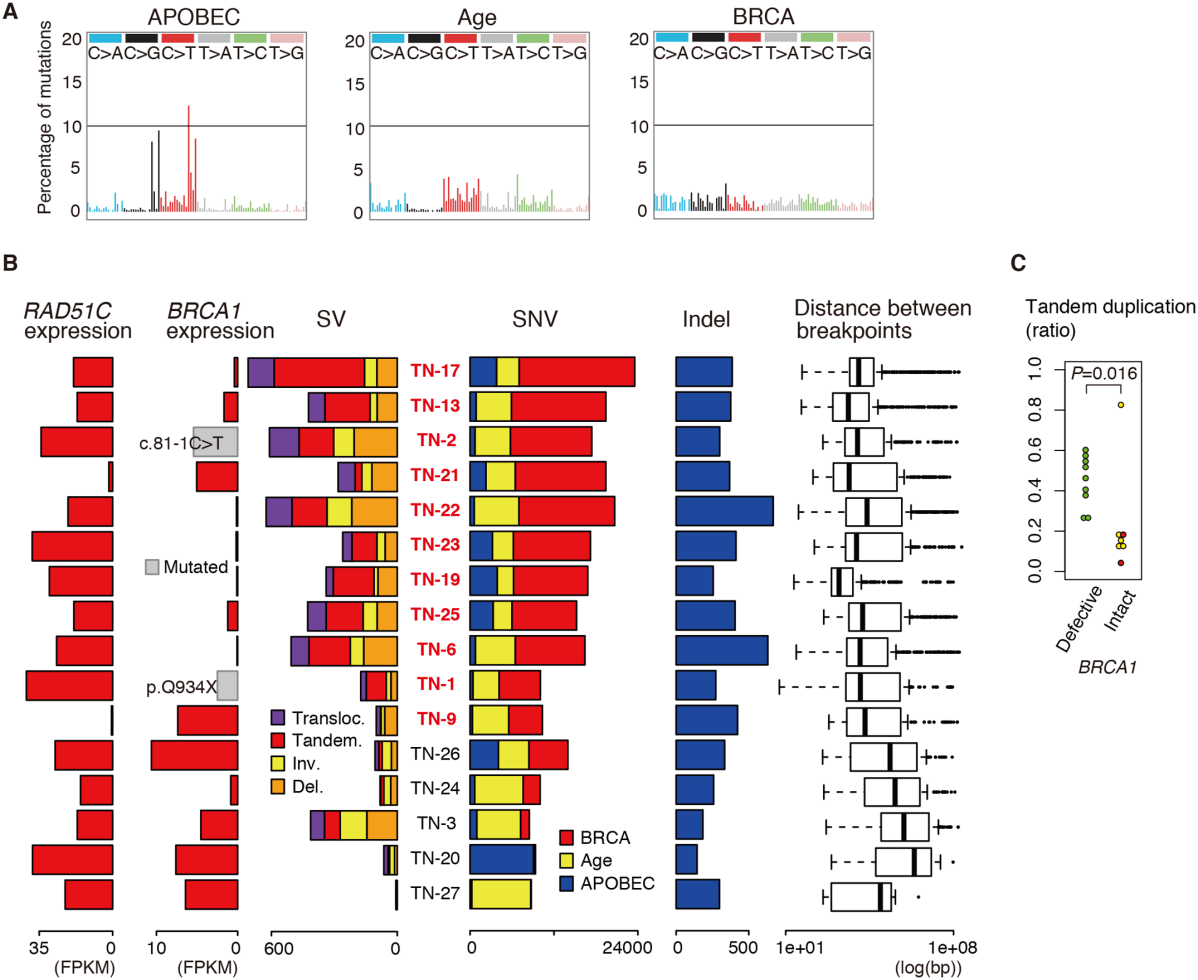
Mutational signatures of triple-negative breast cancer (TNBC). (**A**) Three trinucleotide mutational signatures of single nucleotide variations (SNVs) in whole genome sequencing (WGS) data. (**B**) Numbers of structural variants (SVs), SNVs, and indels in each tumor (middle panel) in association with *RAD51C* and *BRCA1* mRNA expression levels (left panel) and distributions of the distance between breakpoints (right panel). Data are arranged in descending order of *BRCA* signature ratios among SNVs. SNV bars contain both truncal and subclonal SNVs. IDs for samples with a defective homologous recombination (HR) pathway are highlighted in red. *BRCA1* mRNA expression levels in tumors with *BRCA1* mutation are shown in gray. Transloc., translocation; Tandem., tandem duplication; Inv., inverted rearrangement; Del., deletion; FPKM, fragments per kilobase of transcript per million mapped reads. (**C**) Proportions of tandem duplications among all SVs. Green, *BRCA1* silenced or mutated; red, *RAD51C* silenced; yellow, others. The outlier in the *BRCA1* intact group is from TN-27 in which only six SVs were detected.

Mutational signature analysis [21] of SNVs detected by high coverage WGS in 16 TNBC tumors identified the BRCA signature, age-related signature, and APOBEC (apolipoprotein B mRNA editing enzyme, catalytic polypeptide-like) signature (Fig 1A and 1B) that were consistent with previous reports [21–23]. It has been suggested that loss-of-function of several genes, including *BRCA1*, *BRCA2*, *RAD51C*, or *BRIP1*, results in HR deficiency [11,24]. It has also been suggested that the BRCA signature is dominant in tumors with a defective HR pathway [6,24]. In accordance with these notions, HR pathways were expected to be defective in all tumors with a dominant BRCA signature, because of low *BRCA1* or *RAD51C* mRNA expression, or *BRCA1* mutations (Fig 1B). Furthermore, all tumors with low *BRCA1* or *RAD51C* expression exhibited DNA methylation of the corresponding promoter regions (S2 Fig). Thus, we hypothesized that a disruptive mutation and promoter methylation of *BRCA1* or *RAD51C* are the major causes of the defective HR that is manifested by BRCA signature dominance. To validate the relationship between *BRCA1*/*RAD51C* promoter methylation, *BRCA1*/*RAD51C* mRNA expression, and the mutational signature, we analyzed The Cancer Genome Atlas (TCGA) data set (110 TNBC specimens). The three mutational signatures were again extracted from the TCGA exome data (S3 Fig). Low expression of *BRCA1*/*RAD51C* was closely associated with high methylation levels of *BRCA1*/*RAD51C* promoter regions (S3 Fig). When HR defects were defined by promoter methylation or disruptive mutations of *BRCA1*/*RAD51C*, HR defects were significantly associated with the BRCA signature (*P* = 3.2 × 10^−6^, Wilcoxon rank sum test; S3 Fig), supporting our hypothesis and suggesting that mutation and transcriptional silencing of *BRCA1*/*RAD51C* may be determinants of responsiveness to PARP inhibitors.

The high coverage WGS analysis identified a large number of somatic SVs, with a median of 310.5 (range, 6–711) SVs per sample (Fig 1B; S2 Table),which was equivalent to a previous report [6]. They were classified into four types: translocations, inverted rearrangements, deletions, and tandem duplications. In accordance with previous reports [24,25], SV counts were higher in tumors with a defective HR pathway than in tumors with an intact HR pathway (*P* = 0.013, Wilcoxon rank sum test; Fig 1B). Thus, loss of *BRCA1* or *RAD51C* functions was associated with a dominant BRCA signature and higher SV numbers. In our cohort, *BRCA1* deficiency was significantly associated with an increase in the number of tandem duplications (*P* = 0.016, Wilcoxon rank sum test; Fig 1C). Although our cohort contained only two tumors with defective *RAD51C*, it appeared that defective *RAD51C* was not associated with increased tandem duplications. It is thought that a recombination event mediated by break-induced replication (BIR) can lead to the generation of tandem duplication [26]. Because RAD51C facilitates the assembly of RAD51 filaments to promote strand invasion, RAD51C may be required for recombinational DNA repair processes including BIR [27,28]. Further study is required to reveal the contribution of RAD51C to the formation of tandem duplication and the influence of RAD51C deficiency on genomic structural variations.

### Analysis of clonal architectures in TNBC

**Fig 2:**
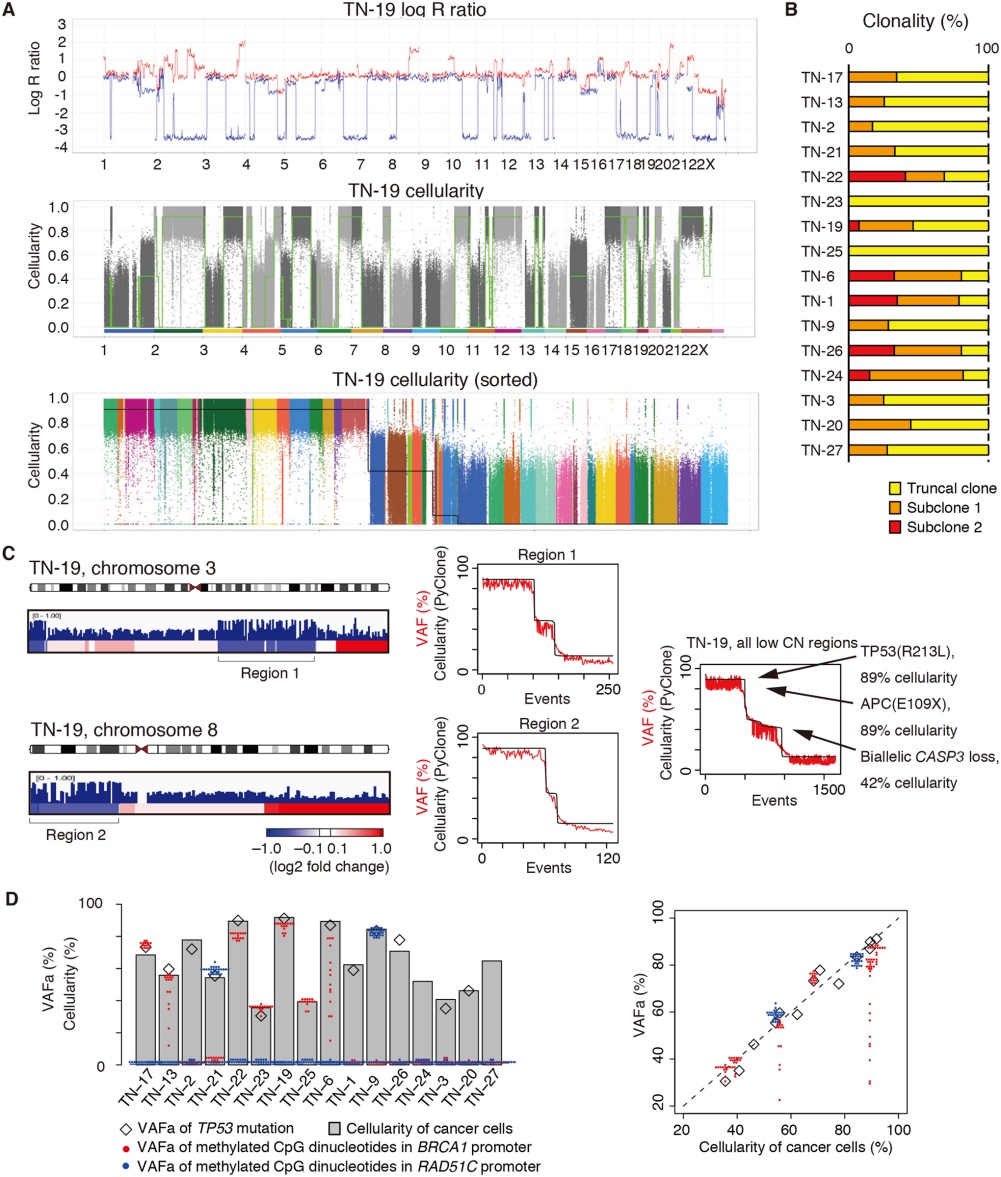
Clonal architecture of triple-negative breast cancer (TNBC). (**A**) CN-based clonal analysis of TN-19. Red and blue lines indicate log R ratios of major and minor alleles, respectively (upper panel). The green line indicates the cellularity of tumor cells harboring the corresponding allelic loss (middle panel). The black line was generated by sorting the plots in the middle panel in descending order (bottom panel). Each dot in the bottom panel represents the single nucleotide polymorphism (SNP) used for the calculation and is color-coded for chromosomes, as indicated in the middle panel. (**B**) Clonality of each subclone in the tumors. To calculate clonality, the cellularity of the truncal clone was set at 100%. (**C**) Single nucleotide variations (SNVs; blue vertical lines) together with the copy number (CN) status of chromosomes 3 and 8 in TN-19. The height of each line represents the variant allele frequency (VAF). CN status is color-scaled: red, gain; blue, loss. Clonal analyses of the indicated regions in TN-19 are shown in the middle: red, observed VAFs; black, cellularity predicted using PyClone. Clonal analysis of VAFs in all low CN regions combined is shown in the right panel. (**D**) Cellularity of each tumor along with the CN-adjusted allele frequency (VAFa) of *TP53* mutations (open diamond) and methylated CpG dinucleotides in the promoter regions of *BRCA1* (red solid circle) and *RAD51C* (blue solid circle). In the right panel, the same data are presented as a scatter plot showing the relationship between cellularity and VAFa of *TP53* mutations (open diamond), or *BRCA1* or *RAD51C* promoter methylation (red and blue solid circles, respectively).

Several sophisticated methods have been used to analyze the clonal architecture of cancer [4,29]. However, the complicated chromosomal copy number (CN) status of TNBC has hampered the precise application of these methods. In particular, the exact number of gained or amplified alleles is quite difficult to determine in the presence of an unknown fraction of contaminating normal cells. Thus, we analyzed clonal architecture using data from regions with a CN of one because the tumor cellularity can be unambiguously determined in such regions. To perform the CN-based global analysis, we first determined the correlation between the minor allele log R ratio and tumor cellularity deduced from the proportion of minor alleles using data from all regions in which the CN of the major allele was one and the CN of the minor allele was zero (S4 Fig). Based on this correlation, the clonal architecture was inferred using data from regions in which the CN of the minor allele was zero (Fig 2A). CN-based global analysis revealed only a few subclones of detectable size in each tumor (Fig 2B), which was consistent with previous results of single cell analysis [30].

We next determined the local clonal architecture of the TNBC samples using variant allele frequencies (VAFs) of SNVs. VAFs within selected regions with low CNs were analyzed separately (Fig 2C, S4 Fig), because the high read depth in the current study enabled such local clonal analysis. In the case of TN-19, analysis using PyClone [4] revealed a truncal clone with 89% cellularity (or 100% clonality by definition) and a subclone with 42% cellularity (or 47% clonality) (Fig 2C, S4 Fig). Of note, this subclone was also identified in the analysis of VAFs within all CN-low regions and the CN-based global analysis (Fig 2A). The truncal clone of TN-19 harbored mutations encoding TP53 (R213L) and APC (E109X), and the major subclone had undergone biallelic loss of a part of the long arm of chromosome 4, encompassing the *CASP3* locus. Analysis of other tumors revealed that the cellularity of cells with *TP53* mutations or methylated CpG dinucleotides in *BRCA1*/*RAD51C* promoter regions coincided with those of truncal clones, suggesting that most *TP53* mutations and the promoter methylation of *BRCA1*/*RAD51C* were acquired in truncal clones (Fig 2D).

The great read depth of the present WGS analysis enabled us to analyze the clonal architecture in detail. The analysis suggested that the CN status of tumors is stable during tumor evolution, and that *TP53* mutations and silencing of *BRCA1*/*RAD51C* are earlier events during TNBC carcinogenesis.

### Characteristics of SVs in TNBC

As previously reported [25], intrachromosomal SVs were more prevalent than interchromosomal SVs (S2 Table). The breakpoints of the intrachromosomal SVs showed that crossover occurred in a complicated manner, indicating the complex nature of the mechanisms underlying SVs in TNBC (S5 Fig). In chromosomal regions where CN changes were prominent, including the long arms of chromosomes 3 and 8, and the short arm of chromosome 6, a large number of SVs were observed (S5 Fig). This finding is not surprising given that CN changes are expected to occur as a result of sequential SVs such as breakage-fusion-bridge cycles [31]. An unexpected observation was that many intrachromosomal SVs crossed over a centromere.

**Fig 3:**
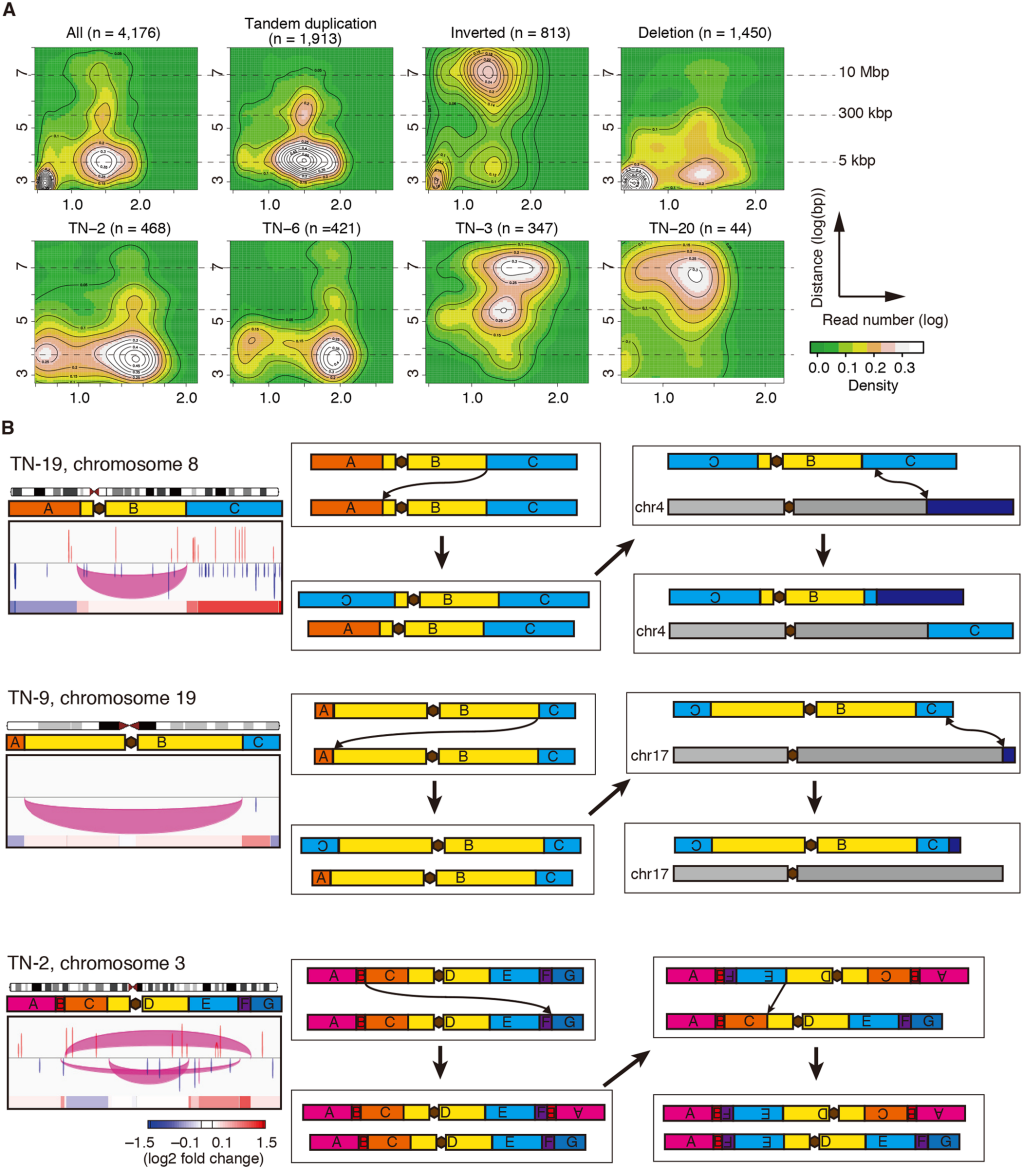
Structural variations (SVs) in triple-negative breast cancer (TNBC). (**A**) Two-dimensional kernel density estimation for the distance between breakpoints and read number. The horizontal axis denotes the supporting read number. The vertical axis denotes the distance between breakpoints. (**B**) Diagrams of chromosome 8 in TN-19, chromosome 19 in TN-9, and chromosome 3 in TN-2, along with SV and copy number (CN) statuses (left). Brown hexagons indicate centromeres. Each pair of break points is connected by a color-coded arch: red, tandem duplication; magenta, inverted rearrangement; blue, deletion. Note that the arches for small SVs appear to be vertical lines owing to limited resolution. CN status is color-scaled: red, gain; blue, loss. Presumed chromosomal rearrangement events are shown in the rectangles in the middle and on the right along with the resultant chromosomes below.

The distances between breakpoints of intrachromosomal SVs had an unexpected trimodal distribution with peaks of approximately 5 kbp, 300 kbp, and 10 Mbp (Fig 3A). Tumors with a defective HR pathway had SVs with short distances between breakpoints (*P* = 4.6E-4, Wilcoxon rank sum test for log_10_(median distance); Figs 1B and 3A). The distances between breakpoints of inverted rearrangements were relatively large with a peak of approximately 10 Mbp, while those of tandem duplications were relatively small with peaks of approximately 5 and 300 kbp. It should be noted that the distance between breakpoints does not always reflect the exact size of the rearrangement because there are many crossovers. More precise assessment of the size of the rearrangement by long read sequencing or phasing (haplotype mapping) technologies in combination with short read sequencing is required in the future.

Large tandem duplications contributed to the CN gains, such as the duplication of the long arm of chromosome 8 in TN-2 and TN-13, and the long arm of chromosome 3 in TN-25 (S5 Fig). Some large inverted rearrangements that crossed over a centromere appeared to result in CN alterations within chromosome 8 in TN-19, chromosome 19 in TN-9, and chromosome 3 in TN-2 (Fig 3B). We proposed a novel mode of CN alterations in which a part of a chromosome arm is replaced by a part of the opposite arm of the homologous chromosome in an inverted rearrangement, thus resulting in CN gain and loss of chromosome regions. Long-range genomic analysis by linked-read sequencing [32] of TN-19 confirmed the break points of the inverted rearrangement in chromosome 8 within consistent haplotype blocks (S5 Fig). The result of three-color fluorescence in situ hybridization (FISH) analysis of the formalin-fixed, paraffin-embedded (FFPE) specimen was compatible with the expected chromosome structure (S5 Fig). Based on this assumption, the observed CN statuses are simply explained by the observed inverted rearrangements (Fig 3B). It would be advantageous for SVs to occur between homologous chromosomes because such an SV does not result in centromere duplication that causes catastrophic mitosis.

In summary, WGS revealed that the size of SVs has a distinctive distribution depending on the type of SVs. It also revealed processes involving inverted rearrangements through which chromosomal regions were gained or lost.

### SVs affect oncogenes and tumor suppressor genes in TNBC

SNVs or indels in putative tumor suppressor genes, such as *RB1*, *KMT2C*, *PTEN*, and *RUNX1*, are relatively infrequent in TNBC compared with ER+ or HER2+ BC [2]. Consistent with this notion, our WES analysis of 36 TNBC tumors identified only two *RB1* mutations, two *KMT2C* mutations, two *PTEN* mutations, and one *RUNX1* mutation. However, our WGS analysis revealed that these tumor suppressor genes were frequently disrupted by SVs in TNBC. Out of 16 analyzed TNBC tumors, six, three, two, and one tumor harbored SVs involving *RB1*, *KMT2C*, *PTEN*, or *RUNX1*, respectively (S1 Fig).

The observed SVs also resulted in the amplification of oncogenes including *MYC*, *NOTCH2*, and *NOTCH3* (S1 Fig). Amplification of the loci resulted in enhanced expression of the respective genes (S6 Fig). We also identified two tumors with amplification of the *NOTCH2* locus by droplet digital PCR CN analysis among 48 FFPE specimens (S6 Fig).

Taken together, these findings indicate that the major consequences of gene rearrangements in TNBC appear to be the disruption of tumor suppressor-coding sequences and the amplification and enhanced expression of oncogenes.

### Recurrent SVs in the putative regulatory region of the *TGFA* gene

**Fig 4:**
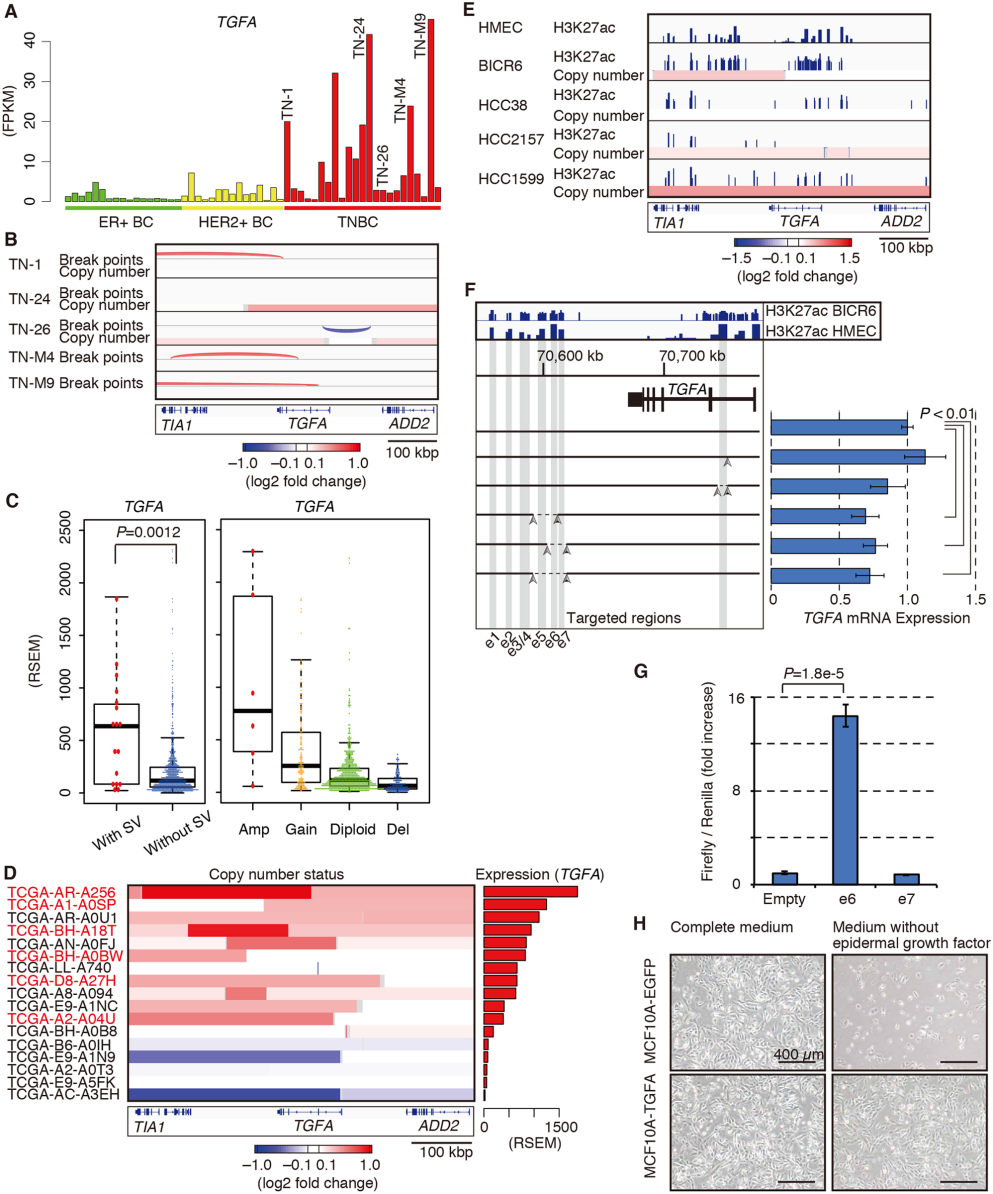
Enhanced expression of *TGFA* is associated with structural variations (SVs) in the putative regulatory region. (**A**) mRNA expression of *TGFA* in ER+ breast cancer (BC) (green), HER2+ BC (yellow), and triple-negative BC (TNBC) (red). IDs for samples with SVs within or near the *TGFA* locus are shown. (**B**) SV break points and copy number (CN) status of samples indicated in (**A**). (**C**) *TGFA* mRNA expression based on The Cancer Genome Atlas (TCGA) data, depending on whether the SV is within or near the *TGFA* locus (left) or according to the CN status (right). (**D**) CN status of BCs, based on TCGA data, with presumed SVs within or near the *TGFA* locus. IDs for TNBC cases are shown in red. (**E**) Acetylation of the lysine residue at position 27 of histone H3 (H3K27ac) detected by ChIP-seq along with the CN status obtained from Cancer Cell Line Encyclopedia (CCLE) data. Data for human mammary epithelial cells (HMECs) were obtained from the Encyclopedia of DNA Elements (ENCODE) project data. (**F**) *TGFA* mRNA expression in BICR6 cell lines in which H3K27ac-enriched putative enhancers (e1–e7) were deleted using the CRISPR-Cas9 system. *P*-values were derived from t-tests. (**G**) Luciferase reporter assays measuring the enhancer activity of e6 and e7 in BICR6 cells. The pGL4.10 plasmid without the enhancer region (empty) was used as a negative control. Relative luciferase units were normalized to Renilla luciferase signals. The normalized value for empty vector was set to 1. *P*-values were derived from t-tests. (**H**) MCF10A cells expressing enhanced green fluorescent protein (EGFP) alone or TGFA together with EGFP, cultured in complete medium or medium without epidermal growth factor.

We further searched for genes that were affected by SVs. SVs in gene regulatory regions influence the expression of genes [33,34]. Whereas *TGFA* expression was activated in a subset of TNBCs (Fig 4A), we observed SVs within or near the *TGFA* locus in five tumors (Fig 4B, S7 Fig). Analysis of CN data from the TCGA BC cohort (1097 specimens including 110 TNBC specimens) revealed 17 possible SVs within or near the *TGFA* locus (Fig 4D), which were enriched in TNBC (6/104 in TNBC, 11/976 in non-TNBC, *P* = 0.0044, Fisher’s exact test). *TGFA* mRNA expression in these tumors was significantly high (*P* = 0.0012, Wilcoxon rank sum test) (Fig 4C and 4D). These data suggested that the observed SVs were associated with enhanced expression of *TGFA*.

*TGFA* expression is elevated in the hypopharyngeal squamous cell carcinoma cell line BICR6 that harbors a focal CN gain involving the *TFGA* locus, similar to that of TNBC tumor samples (TN-M4 in the present study and TCGA-AR-A256 in TCGA), according to Cancer Cell Line Encyclopedia (CCLE) data (Fig 4B, 4D and 4E). Acetylation of the lysine residue at position 27 of histone H3 (H3K27ac) was enriched on the duplicated *TGFA* locus in BICR6 cells (Fig 4E). Although the BICR6 cell line does not originate from mammary tissue, the H3K27ac profile of the *TGFA* locus in BICR6 cells was similar to that in human mammary epithelial cells according to the Encyclopedia of DNA Elements (ENCODE) project data. The binding profile of H3K27ac identified seven putative enhancers (e1–e7; Fig 4F), among which e6 was the most prominently enriched for H3K27ac (S7 Fig). These observations indicated that the H3K27ac-enriched regions might be regulatory regions of *TGFA* expression, and that BICR6 could be used to investigate the functional role of SVs within or near the *TGFA* locus.

To determine whether the H3K27ac-enriched genomic regions were required for the enhanced expression of *TGFA* in BICR6 cells, the regions encompassing e6 were deleted using the CRISPR-Cas9 system. Deletion of the regions in BICR6 cells resulted in decreased expression of *TGFA* (Fig 4F), indicating a direct regulatory function of the region. In luciferase reporter assays using BICR6 cells, the e6 region was found to have strong activity (Fig 4G). In contrast, ectopic expression of *TGFA* conferred growth factor independence on MCF10A immortalized mammary epithelial cells (Fig 4H). Taken together, these results suggested that SVs involving putative regulatory regions of *TGFA* in TNBC could result in enhanced expression of *TGFA*, another candidate oncogene in TNBC.

### Various oncogenic driver mutations in TNBC

**Fig 5:**
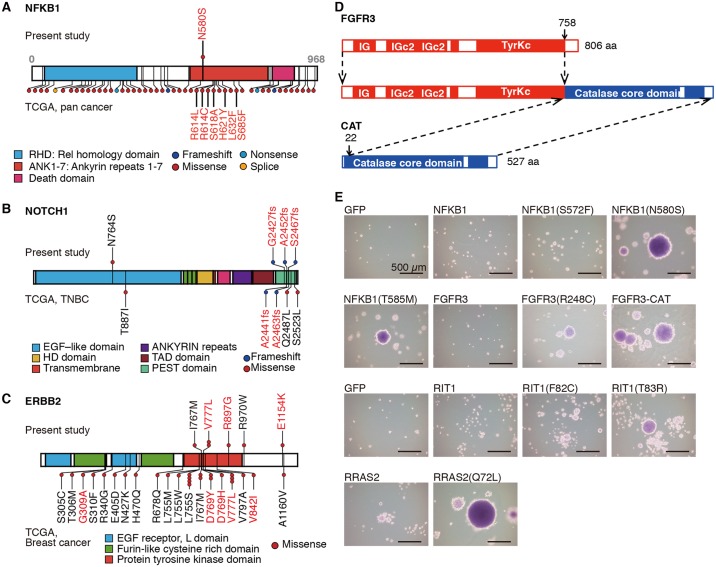
Various oncogenes in triple-negative breast cancer (TNBC). (**A**) NFKB1 mutations found in this study (above, TNBC) and in the TCGA data (below, various cancers). (**B**) Mutations in NOTCH1 found in this study (above, TNBC) and in the TCGA data (below, TNBC). (**C**) Mutations in ERBB2 found in this study (above, TNBC) and in the TCGA data (below, BC). (**D**) Diagram of the protein encoded by the *FGFR3*-*CAT* fusion gene. IG, immunoglobulin-like domain; TyrKc, tyrosine-protein kinase catalytic domain. (**E**) Anchorage-independent growth of 3T3 cells expressing the indicated proteins. The diagrams of the proteins in (**A**), (**B**), and (**C**) were generated using protein painter (http://explore.pediatriccancergenomeproject.org/proteinPainter). Mutations expected to be activating according to previous reports or experiments in this study are highlighted in red.

Next, we searched for SNVs that produce potential oncogenes by the soft agar assay, a classical biological assay using the 3T3 immortalized mouse fibroblast cell line [17]. We identified a mutation in the *NFKB1* gene encoding NFKB1 (N580S) (Fig 5A). According to TCGA pan-cancer data, mutations in *NFKB1* are scattered along the protein with moderate enrichment within the ankyrin repeat domain (Fig 5A), but the mutations are quite infrequent. Although another mutation in the ankyrin repeat domain of NFKB1 (T585M) has been detected in a BC specimen [29], its significance has not been analyzed. Remarkably, both NFKB1 (N580S) and (T585M) conferred anchorage-independent growth on mouse 3T3 fibroblasts (Fig 5E), strongly suggesting the oncogenic potential of the mutant proteins. p50, the active subunit of the transcription factor NF-κB, is processed from full length NFKB1 or p105. Full length NFKB1 (p105) tethers p50 within the cytoplasm and prevents nuclear translocation of p50 [35]. Biochemical analyses indicated that the affinity of these NFKB1 mutants for p50 was lower than that of wild-type NFKB1, thus leading to increased nuclear translocation of p50 (S8 Fig), which in turn leads to *CCND1* activation [36].

Two out of 36 frozen tumor samples harbored frame shift mutations within the PEST domain of NOTCH1 (Fig 5B), which are well-described activating mutations of NOTCH1 in T-cell acute lymphoblastic leukemia [37]. One NOTCH1 PEST domain mutation was found among 48 FFPE samples. By combining the three tumors with *NOTCH2* locus amplification and a tumor with *NOTCH3* locus amplifications, seven tumors with an activated NOTCH pathway were identified out of 84 tumors. These data indicated that the NOTCH pathway was genetically altered and activated in a subset of TNBCs, which is in agreement with a previous report [14].

Activating mutations of *ERBB2* have been shown to contribute to carcinogenesis [38]. Two out of 36 frozen tumor samples harbored such *ERBB2* mutations (Fig 5C). In addition, two out of 48 FFPE TNBC tumors and two out of 31 ER+ tumors harbored *ERBB2* mutations (S3 Table). *ERBB2* mutations were enriched in HER2+ BCs compared with ER+ BCs and TNBCs in the TCGA BC data (*P* = 0.00061, S4 Table).

We also identified mutations in small GTPases, such as RIT1 (T83R), RRAS2 (Q72L), and MRAS (R83H), in TNBC samples. RIT1 (T83R) and RRAS2 (Q72L) were found to be oncogenic in a soft agar assay (Fig 5E, S9 Fig), whereas MRAS (R83H) did not show a transforming capacity in a 3T3 transformation assay. Although no *RIT1*, *RRAS2*, or *MRAS* mutations were found in the TCGA BC dataset, one tumor with undetermined ER and HER2 statuses harbored a RAC1 (P69S) mutation, and one TNBC tumor harbored a RHOB (D13Y) mutation. Both of these mutant proteins were confirmed to be oncogenic in a tumorigenicity assay in nude mice (S9 Fig).

We also identified one tumor that expressed an *FGFR3*-*CAT* fusion transcript (Fig 5D). 3T3 cells expressing the FGFR3-CAT fusion protein formed tumors in mice and exhibited anchorage-independent growth in a soft agar assay (Fig 5E, S9 Fig). Thus, SV in TNBC resulted in oncogenic gene fusion. However, our attempt to identify fusion genes involving *FGFR* genes (*FGFR1*, *FGFR2*, and *FGFR3*) in the TCGA RNA-seq data was unsuccessful.

The identification of the wide variety of oncogenic driver events presented above suggested that individual TNBC tumors might harbor a unique oncogenic driver event that is not always found in TNBC.

## Discussion

In the present study, through high coverage WGS, we comprehensively analyzed the genetic alterations in TNBC. We have presented some novel findings about the genetic features of TNBC. Furthermore, we searched for actively functioning oncogenes, because identification of oncogenes has the potential to contribute to the development of targeted therapies.

First, our analysis focused on the genetic features of tumors with HR deficiency. Our results were in accordance with the recently developed notion that tumors with a defective HR pathway harbor SNVs with the BRCA signature, relatively abundant SVs, and relatively abundant tandem duplications [6,24]. We showed that tumors with *BRCA1* or *RAD51C* promoter methylation exhibit a similar molecular phenotype to those with *BRCA1* mutations. It should be clarified whether these tumors respond to platinum-based antitumor agents and PARP inhibitors in a similar manner to tumors with *BRCA1* mutations in future studies. In this respect, it is noteworthy that the cellularity of cells with methylated CpG dinucleotides in *BRCA1* promoter regions of the TN-6 and TN-13 samples were variegated, while those in other specimens coincided with tumor cellularity (Fig 2D). This observation indicated that the promoter of *BRCA1* was not fully methylated in a subset of tumor cells in TN-6 and TN-13 samples, and that these cells may develop resistance to platinum-based antitumor agents or PARP inhibitors.

Second, our data indicated the evolutionary pathway of TNBC carcinogenesis in which HR deficiency plays a pivotal role. It is reasonable to consider that *TP53* and the HR pathway are impaired at the initial phase of TNBC carcinogenesis, causing the generation of SVs, which in turn disrupts the coding sequences of other tumor suppressors, such as *RB1*, *KMT2C*, and *PTEN*, and cause amplification of the *MYC* oncogene, as proposed previously [6,18]. Accordingly, to reveal the pathogenesis of TNBC and identify the ideal therapeutic target, more attention should be focused on SVs that affect not only protein-coding sequences, but also noncoding regulatory elements.

Third, we searched for oncogenes that positively regulate the proliferation of tumor cells using biological assays for cellular transformation where possible. We found that SVs encompassing putative regulatory regions of *TGFA* were associated with high expression of *TGFA*. Because ectopic expression of TGFA confers growth factor independence on MCF10A cells, and the oncogenic potential of TGFA has been demonstrated using a transgenic mouse model [20], it is expected that TGFA can be a therapeutic target. Further study is required to reveal the contribution of TGFA to the proliferation and survival of TNBC. In addition, we identified various oncogenic gene alterations, some of which may be targetable. Although the frequency of each identified event was rare, identification of potent oncogenes has the potential to provide important information for treatment options for patients whose tumors harbor that particular oncogene.

The high coverage WGS integrated with exome sequencing and RNA-seq in the present study revealed several important aspects of TNBC biology and also yielded data that are potentially useful in the era of clinical sequencing and personalized medicine.

## Methods

### Human research ethics approval

The genomic analysis of primary tumor tissue samples was approved by the Human Genome, Gene Analysis Research Ethics Committee of The University of Tokyo.

### Library preparation for WGS, WES, and RNA-seq

Genomic DNA was isolated from each sample and prepared for WGS with an NEBNext Ultra DNA Library Prep Kit (New England BioLabs, Ipswich, MA) according to the manufacturer’s instructions. Adaptor-ligated samples were amplified by three PCR cycles. For WES, genomic DNA was subjected to enrichment of exonic fragments with a SureSelect Human All Exon Kit v5 (Agilent Technologies, Santa Clara, CA). cDNA was prepared from isolated RNA using an NEBNext Ultra Directional RNA Library Prep Kit (New England BioLabs). Massively parallel sequencing of the prepared samples was performed with a HiSeq2000/2500 platform (Illumina, San Diego, CA) using the paired-end option.

### Analysis of WGS data

Paired-end reads of WGS were aligned to the human reference genome (hg19) using the Burrows-Wheeler Aligner (BWA, http://bio-bwa.sourceforge.net/) [39]. SNVs, indels, and SVs were called using our in-house program as described previously [40,41] with some modification.

To predict somatic SNVs and indels, the filters described previously were applied. SNVs and indels were selected when the frequency of the non-reference allele was at least 5% in the tumor genome. In our somatic mutation call, we first compared variants in a matched pair (tumor/normal sample for each individual patient) and removed personal germline variants. Next, we made a comparison with all normal samples grouped together, a so-called “normal panel”, and removed false positive variants that occurred by sequence errors. This strategy is very effective for removing false positives because sequence errors occur in a sequence-specific manner at a certain frequency rather than randomly.

Fifty base-pair paired-end reads were used for rearrangement analysis, because they contain longer spacers than 125 bp paired-end reads. Therefore, 125 bp paired-end reads were separated to generate 50 bp paired-end reads. To detect structural variations, we used a paired-end read for which both ends aligned uniquely to the human reference genome, but with improper spacing, orientation, or both.

First, paired-end reads were selected based on the following filtering conditions: (i) sequence read with a mapping quality score greater than 37; (ii) sequence read aligned with two mismatches or less. Rearrangements were then identified using the following analytical conditions: (i) forward and reverse clusters, which included paired-end reads, were constructed from the end sequences aligned with forward and reverse directions, respectively; (ii) two reads were allocated to the same cluster if their end positions were not farther apart than 400 bp; (iii) paired-end reads were selected if one end sequence fell within the forward cluster and the other fell within the reverse cluster (we hereafter refer to this pair of forward and reverse clusters as paired-clusters); (iv) for the tumor genome, rearrangements predicted from paired-clusters, which included at least six pairs of end reads, were selected; (v) rearrangements detected in the tumor genome, but not present in the panel of non-tumor genome (all non-tumor genomes grouped together), were selected as somatically acquired rearrangements.

### Analysis of WES data

Paired-end WES reads were aligned to the human reference genome (hg19) using BWA [39], Bowtie2 (http://bowtie-bio.sourceforge.net/bowtie2/index.shtml) [42], and NovoAlign (http://www.novocraft.com/products/novoalign/) independently. Somatic mutations were called using MuTect (http://www.broadinstitute.org/cancer/cga/mutect) [43], SomaticIndelDetector (http://www.broadinstitute.org/cancer/cga/node/87) [44], and VarScan (http://varscan.sourceforge.net) [45]. Mutations were discarded if (1) the read depth was <20 or the variant allele frequency (VAF) was <0.1, (2) they were supported by only one strand of the genome, or (3) they were present in the “1000 genomes” database (http://www.1000genomes.org) or in normal human genomes from our in-house database. Gene mutations were annotated by SnpEff (http://snpeff.sourceforge.net) [46]. CN status was analyzed by our in-house pipeline that calculates the log R ratio using normal and tumor VAFs based on dbSNPs of the 1000 genomes database.

### Analysis of RNA-seq data

For expression profiling with RNA-seq data, paired-end reads were aligned to the hg19 human genome assembly using TopHat2 (https://ccb.jhu.edu/software/tophat/index.shtml) [47]. The expression level of each RefSeq gene was calculated from mapped read counts using Cufflinks (http://cufflinks.cbcb.umd.edu) [48].

### Analysis of mutational signatures and the clonal architecture

Mutational signatures were analyzed using the Wellcome Trust Sanger Institute Mutational Signature Framework (http://jp.mathworks.com/matlabcentral/fileexchange/38724-wtsi-mutational-signature-framework) [49]. The optimal number of signatures was determined according to signature stabilities and average Frobenius reconstruction errors.

CN-based clonal analysis was conducted as follows: (1) we first chose genomic regions where the CN of the major allele was one and the CN of the minor allele was zero; (2) at each selected region, the cellularity of tumor cells harboring the CN alteration was deduced from the minor allele proportion; (3) correlations between the minor allele log R ratio (minor allele LRR) and tumor cellularity were determined; (4) by k-means clustering of the minor allele LRR values of each sample, minor allele LRR values representing the tumor clone and subclones were inferred; (5) cellularity values were calculated using the above correlation of (3) and representative value of (4). Clonality was then determined by setting the cellularity of the truncal clone to 100%. For the clonal analysis using VAFs at low CN regions, PyClone (http://compbio.bccrc.ca/software/pyclone/) [50] was used.

### Molecular barcoding of high molecular weight genomic DNA

Genomic DNA of TN-19 was analyzed to obtain long-range genomic information by molecular barcoding [32]. Partition barcoded libraries with sample indexing were prepared on a Chromium Controller Instrument (10X Genomics, Pleasanton, CA) using a Chromium Genome Reagent Kit (10X Genomics) according to manufacturer’s protocols. Sequencing of the prepared samples was performed with a HiSeq2500 platform (Illumina) using the paired-end option (2 × 130 paried-end reads). Data were analyzed with Long Ranger (10X Genomics, https://support.10xgenomics.com/genome-exome/software/pipelines/latest/what-is-long-ranger) and visualized with Loupe (10X genomics, https://support.10xgenomics.com/genome-exome/software/visualization/latest/what-is-loupe).

### TCGA and CCLE data acquisition

To analyze mutational signatures, mRNA expression and methylation, level 2 mutation data, level 3 mRNA expression data (RNA-seq V2 RSEM) and level 3 methylation data from the TCGA invasive breast carcinoma cohort were obtained from the TCGA data portal. For the mutational analysis of specific genes, including *NOTCH1*, *ERBB2*, and genes encoding small GTPases, level 1 sequencing data in BAM format were downloaded via The Cancer Genomics Hub. Data from 906 tumor-normal pairs were subjected to mutation calling with MuTect. To detect fusion transcripts, level 1 prealigned RNA-seq data in BAM format were downloaded from The Cancer Genomics Hub. To determine ER and HER2 statuses, clinical information was downloaded from the TCGA data portal. CCLE CN data were downloaded from the Memorial Sloan Kettering Cancer Center’s cBio portal [51]

### Bisulfite sequencing

Genomic DNA was subjected to bisulfite conversion with an EpiTect Bisulfite Kit (Qiagen, Valencia, CA). Converted DNA fragments were amplified by PCR using a Kapa HiFi Uracil+ Kit (Kapa Biosystems, Woburn, MA) with the following primer sets:

*BRCA1*-1-S, 5′-TTAGAGTAGAGGGTGAAGGTTTTTT-3′;

*BRCA1*-1-AS, 5′-AACAAACTAAATAACCAATCCAAAAC-3′;

*BRCA1*-2-S, 5′-TTTTTTAGTTTTAGTGTTTGTTATTTTT-3′;

*BRCA1*-2-AS, 5′-CCAAACTACTTCCTTACCAACTTC-3′;

*BRCA1*-3-S, 5′-GTTGGTAAGGAAGTAGTTTGGGTTAG-3′;

*BRCA1*-2-AS, 5′-AAACTCTCTCATCCTATCACTAAAAC-3′;

*RAD51C*-1-S, 5′-GTTGAGGAATTTTTAGAGGTGAAATT-3′;

*RAD51C*-1-AS, 5′-ATTCAAACAACTTATAAATAAAATC-3′;

*RAD51C*-2-S, 5′-GAGAATTTATTGGGTTTGGTTTTT-3′;

*RAD51C*-2-AS, 5′-AATTTCACCTCTAAAAATTCCTCAAC-3′. Amplified PCR products were prepared for high throughput sequencing.

### Three-color FISH analysis

FFPE 4 μm-thick sections were treated using an FFPE FISH Pretreatment Kit (GSP Laboratory, Kobe, Japan) and hybridized with BAC clone-derived three-color probes in a humidified chamber overnight at 37°C. Texas red-, Cy5-, and FITC-labeled probes were designed as the three-color probes to detect *FGFR1*, *RUNX1T1*, and *CCNE2* loci, respectively (GSP Laboratory). The sections were washed in 2× SSC, counterstained with 4,6-diamidino-2-phenylindole, and observed under a fluorescence microscope (Leica CTR6000; Leica Microsystems, Wetzlar, Germany).

### Cell lines

Human embryonic kidney (HEK) 293T cells and mouse 3T3 fibroblasts were obtained from the American Type Culture Collection and maintained in Dulbecco’s modified Eagle’s medium (DMEM)-F12 supplemented with 10% fetal bovine serum (FBS) (both from Life Technologies, Carlsbad, CA). The hypopharynx squamous cell carcinoma cell line BICR6 was obtained from Sigma-Aldrich and maintained in DMEM-F12 supplemented with 10% FBS.

### ChIP-seq analysis

ChIP was performed using a SimpleChIP Plus Enzymatic Chromatin IP Kit (Cell Signaling Technology, Danvers, MA) according to the manufacturer’s instructions. Briefly, crosslinking of chromatin was achieved by incubation in 1% formaldehyde at room temperature for 10 min. Crosslinked chromatin was fragmented enzymatically with MNase for 25 min at 37°C. The antibodies used for ChIP were as follows: negative control normal rabbit IgG antibody (Cell Signaling Technology, #2729), anti-histone H3 rabbit mAb (#4620), and anti-acetyl-histone H3 (Lys27) rabbit mAb (#8173). After reversal of crosslinking of immunoprecipitated chromatin, genomic DNA was extracted and then prepared for high throughput sequencing.

### Deletion of genomic regions using the CRISPR-Cas9 system

LentiCas9-Blast (Addgene plasmid # 52962) was a gift from Feng Zhang (Broad Institute, Cambridge), and pgRNA-humanized (Addgene plasmid # 44248) was a gift from Stanley Qi (Stanford University, Stanford) [52,53]. The following target sequences for guide RNAs were cloned into pgRNA-humanized: *TGFA*-int1-2, 5′-CCCTGGGGTATACCTGTGAG-3′; *TGFA*-int1-6, 5′-GGGTCACTCCAAACAAAGGA-3′; *TGFA*-en-4, 5′-CCTGATGAGCATACACTCCG-3′; *TGFA*-en-5, 5′-TATTCTCTCGGTCCTGCACG-3′; *TGFA*-en-6, 5′-CACCTTAGGTACCAGCCGTG-3′; *TGFA*-en-7, 5′-TGTATCTAGCACTTAGACCA-3′. BICR6 cells were infected with LentiCas9-Blast, followed by selection with 10 μg/ml blasticidin for 4 days. BICR6 cells stably expressing Cas9 were then infected with a pair of pgRNAs to achieve deletion of the indicated genomic regions.

### Luciferase reporter assay

The pGL4.10 luciferase vector (Promega, Madison, WI) was used. The enhancer regions were cloned upstream of the luciferase-coding sequence. The reporter constructs were then cotransfected with a control pGL4.74 (Promega) vector expressing Renilla luciferase. The luciferase signal was first normalized to the Renilla luciferase signal and then normalized to the signal of the empty pGL4.10 plasmid. Primers used for cloning were as follows: e6-S, 5′-TGTATGGGTTTCTTCCTGGGCTGT-3′; e6-AS, 5′-CAGTTTTTCAGGTTTCTCTGGGGTCC-3′; e7-S, 5′-TGGGCTTCATGACAGCATCCCTA-3′; e7-AS, 5′-TTGACATGGGCCATTACTCCATCC-3′.

### Colony formation in soft agar and the tumorigenicity assay in nude mice

The coding sequences of genes were amplified by RT-PCR and inserted into the retroviral plasmid pMXs-ires-EGFP (Clontech, Mountain View, CA). To produce infectious viral particles, HEK293T cells were transfected with the plasmids together with ecotropic retroviral packaging plasmids (Takara Bio, Otsu, Shiga, Japan). Virus particles were then used to infect 3T3 cells that were subsequently suspended in culture medium containing 0.4% (wt/vol) agar (SeaPlaque GTG agarose; FMC BioProducts, Rockland, ME) and layered on top of culture medium containing 0.53% (wt/vol) agar in six-well plates. Colonies were allowed to form for 21 days and then stained with crystal violet. 3T3 cells (1 × 10^6^) expressing wild-type or mutant forms of the indicated proteins were also injected subcutaneously into BALB/c nu/nu mice for *in vivo* tumorigenicity assays. The mouse experiments were approved by the Institutional Animal Care and Use Committee of the University of Tokyo.

### Functional analysis of mutant forms of NFKB1

The coding sequences of wild-type and mutant forms of *NFKB1* were amplified by RT-PCR and inserted into the expression vector pcDNA3. The plasmids were transfected into HEK293T cells, and then nuclear translocation of the N-terminal half of NFKB1 protein (p50) was analyzed as follows. Cytoplasmic and nuclear fractions were prepared from cell lysates, separated by SDS-polyacrylamide gel electrophoresis, transferred to a membrane, and then subjected to western blotting to detect p50 and full-length NFKB1 protein (p105) using an anti-NFKB1 antibody (Cell Signaling Technology #3035). The coding sequences of the C-terminal region (CTR) of wild-type and mutant forms of NFKB1, which were tagged with Myc peptide, were amplified by RT-PCR and inserted into the expression vector pcDNA3. The plasmids were transfected into HEK293T cells along with a plasmid expressing p50 tagged with the FLAG peptide. Total cell lysates were immunoprecipitated with an anti-FLAG antibody (M2, Sigma-Aldrich, St. Louis, MO) and analyzed using an anti-Myc antibody (Cell Signaling Technology, #2276). To assess the stability of wild-type and mutant forms of the CTR, transfected HEK293T cells were treated with 10 μg/ml cycloheximide for the indicated times before total cell lysates were prepared. The total cell lysates were analyzed with anti-FLAG and anti-Myc antibodies. The densities of detected bands were measured using ImageJ software (https://imagej.nih.gov/ij/).

### Accession number

The raw sequencing data have been deposited in the Japanese Genotype-Phenotype Archive (JGA, http://trace.ddbj.nig.ac.jp/jga), which is hosted by DDBJ, under accession number JGAS00000000095.

## Supporting information

S1 Fig Summary of the somatic mutations identified in triple-negative breast cancer (TNBC). (**A**) Numbers of single nucleotide variations (SNVs) identified by whole exome sequencing (WES). Data are arranged in descending order of the BRCA signature SNV ratio. IDs of samples subjected to whole genome sequencing (WGS) are indicated by a cyan shadow. Mutations of well-known tumor suppressors and driver oncogenes are shown below. Mutations identified by WGS are also included: yellow, SNV and indel; red, structural variation (SV); orange, copy number gain or amplification. (**B**) Variant allele frequencies (VAFs) of *TP53* mutations among RNA-seq reads plotted against VAFs among WES reads.
(TIF)

S2 Fig Silencing of *BRCA1* and *RAD51C*. The methylation status of CpG dinucleotides in each patient, as assessed by bisulfite sequencing. The proportion of methylated alleles at each cytosine residue is presented as a vertical line. Red bars indicate analyzed regions. Blue lines indicate exon 1 of *BRCA1* (**A**) and *RAD51C* (**B**).
(TIF)

S3 Fig Analysis of the mutational signatures of single nucleotide variations (SNVs) in The Cancer Genome Atlas (TCGA) triple-negative breast cancer (TNBC) data. (**A**) Three trinucleotide mutational signatures identified by analysis of SNVs. (**B**) Numbers of SNVs in association with mRNA expression and promoter methylation of *BRCA1* and *RAD51C*. Data are arranged in descending order of BRCA signature SNV ratios. Expression status is color-scaled: blue, low. Methylation status is color-scaled: red, high; gray, no data. Data from probes cg19088651 (*BRCA1*), cg27253386 (*BRCA2*), and cg02118635 (*RAD51C*) are shown. (**C**) mRNA expression of *BRCA1* and *RAD51C* plotted against methylation levels. The threshold for probes cg19088651 and cg02118635 was set to 0.2. (**D**) Proportions of BRCA signatures. It was assumed that the homologous recombination (HR) pathway was defective when the *BRCA1* (cg19088651) or *RAD51C* (cg02118635) methylation β value was more than 0.2 or *BRCA1* harbored a deleterious somatic mutation. Information about germline mutations was not available.
(TIF)

S4 Fig Analysis of clonal architecture. (**A**) Tumor cellularity deduced from the minor allele proportion plotted against minor allele Log R ratios at all regions where the copy number (CN) of the major allele was one and the CN of the minor allele was zero. (**B**) Red and blue lines indicate the log R ratios of major and minor alleles, respectively (upper panel). Single nucleotide variations (SNVs; blue vertical lines) are shown along with the CN status (middle panels). The height of each line represents the variant allele frequency (VAF). Clonal analysis of selected regions where one allele was lost is shown (lower panels): red, observed VAFs; black, cellularity predicted using PyClone.
(TIF)

S5 Fig Status of intrachromosomal rearrangements. (**A**) Structural variants (SVs) along with the copy number (CN) status. Each pair of break points constituting an SV is connected by a color-coded arch: red, tandem duplication; magenta, inverted rearrangement; blue, deletion. Note that arches for small SVs appear to be vertical lines owing to limited resolution. The CN status is color-scaled: red, gain; blue, loss. Chromosomes 1, 3, 6, and 8 are shown as representative examples. (**B**) The status of chromosome 8 in TN-13 as a representative example of a high resolution image. (**C**) Three-color fluorescence in situ hybridization (FISH) analysis of the TN-19 specimen. *FGFR1*, *RUNX1T1*, and *CCNE2* loci were detected with Texas red-, Cy5-, and FITC-labeled probes, respectively. Representative high power fields are shown. Presumed structures of chromosome 8 in TN-19 are shown schematically in the upper panel. Brown hexagons indicate centromeres. Solid circles indicate the probes: magenta, *FGFR1*; cyan, *RUNX1T1*; green, *CCNE2*. Colored arrowheads indicate combinations of adjacent probes. (**D**) Validation of the inverted rearrangement in TN-19 by linked-read sequencing. Heat map of overlapping barcodes plotted for inverted rearrangement on chromosome 8 in TN-19 is shown (bottom panel). Linearized view of the barcode overlap matrix is also shown (upper panel).
(TIF)

S6 Fig Activation of the NOTCH pathway in triple-negative breast cancer (TNBC). (**A–C**) mRNA expression of *MY*C (**A**), *NOTCH2* (**B**), and *NOTCH3* (**C**) in ER+ breast cancer (BC) (green), HER2+ BC (yellow), and TNBC (red) are shown (left panel) along with the copy number (CN) status of TNBC samples analyzed by whole genome sequencing (right panel). Horizontal blue lines indicate gene loci. The CN status is color-scaled: red, gain; blue, loss. (**D**) CN values of *NOTCH2* and *NOTCH3* estimated by droplet digital PCR in 48 FFPE samples.
(TIF)

S7 Fig Breakpoints of structural variations (SVs) associated with putative *TGFA* regulatory regions. (**A**) Breakpoints associated with tandem duplications near the *TGFA* locus were amplified by PCR of genomic DNA from patients TN-1, TN-M4, and TN-M9, followed by Sanger sequencing analysis. (**B**) Acetylation of the lysine residue at position 27 of histone H3 (H3K27ac) in BICR6 cells detected by ChIP-seq. Putative TGFA regulatory regions are indicated (e1–e7).
(TIF)

S8 Fig Biochemical analysis of mutant forms of NFKB1. (**A**) Mouse 3T3 cells were infected with an empty retrovirus (Mock) or recombinant retrovirus encoding either wild-type or mutant forms of NFKB1. Cytoplasmic (left panel) and nuclear (right) fractions of these cells were prepared and subjected to immunoblot analysis with antibodies against p105/p50, lamin B, or β-actin, as indicated. (**B**) HEK293T cells were transfected with a vector encoding either wild-type or mutant forms of the C-terminal region of NFKB1 tagged with the Myc peptide (CTR-Myc) along with an empty expression vector (–) or a vector encoding FLAG-tagged p50 (+). Total cell lysate (TCL) extracted from transfected cells was subjected to immunoprecipitation (IP) with an anti-FLAG antibody. TCL and immunoprecipitated fractions were analyzed using antibodies against Myc peptide and FLAG peptide. (**C**) HEK293T cells were transfected with a mock vector or a vector encoding either wild-type or mutant forms of CTR-Myc. TCL was subjected to immunoprecipitation with an anti-Myc antibody and then subjected to intensive washing. Purified wild-type and mutant forms of CTR-Myc were mixed with recombinant p50 (INPUT). CTR-Myc was pulled down and analyzed by immunoblotting. (**D**) HEK293T cells were transfected with a vector encoding either wild-type or mutant forms of CTR-Myc along with an empty expression vector (–) or a vector encoding FLAG-tagged p50 (+). Cells were treated with 10 μg/ml cycloheximide (CHX) to inhibit protein synthesis for the indicated duration. TCL was analyzed by immunoblotting to assess the stability of proteins. The amount of CTR-Myc measured by densitometry is shown below. GFP was used as a loading control from which the density of CTR-Myc was calculated. Compensated values of CTR-Myc in the absence of p50 at 0 h are set to 1: CTR-Myc with p50, white squares; CTR-Myc without p50, black squares.
(TIF)

S9 Fig Tumorigenic potential of mutant forms of small GTPases. 3T3 cells expressing wild-type or mutant forms of the indicated proteins were injected subcutaneously into the shoulders of nude mice. (**A**) Representative images of tumors at the indicated times. The numbers of generated tumors (number of generated tumors/number of injection sites) are indicated. (**B**) Tumor sizes [(length × width)] at the indicated times. Data are the means ± standard deviation.
(TIF)

S1 Table Sequencing applications applied to BC samples. (XLSX)

S2 Table Statistics obtained with whole-genome sequencing of 16 cases of triple-negative breast cancer. (XLSX)

S3 Table *ERBB2* mutations detected in the present study. (XLSX)

S4 Table *ERBB2* mutational status of TCGA BC specimens. (XLSX)
