# Supplementary figures and images for: Integrative analysis of genomic alterations in triple-negative breast cancer in association with homologous recombination deficiency
Source: PLoS Genet. 2017 Jun 21;13(6):e1006853. doi: 10.1371/journal.pgen.1006853 (PMC5500377; doi:10.1371/journal.pgen.1006853)

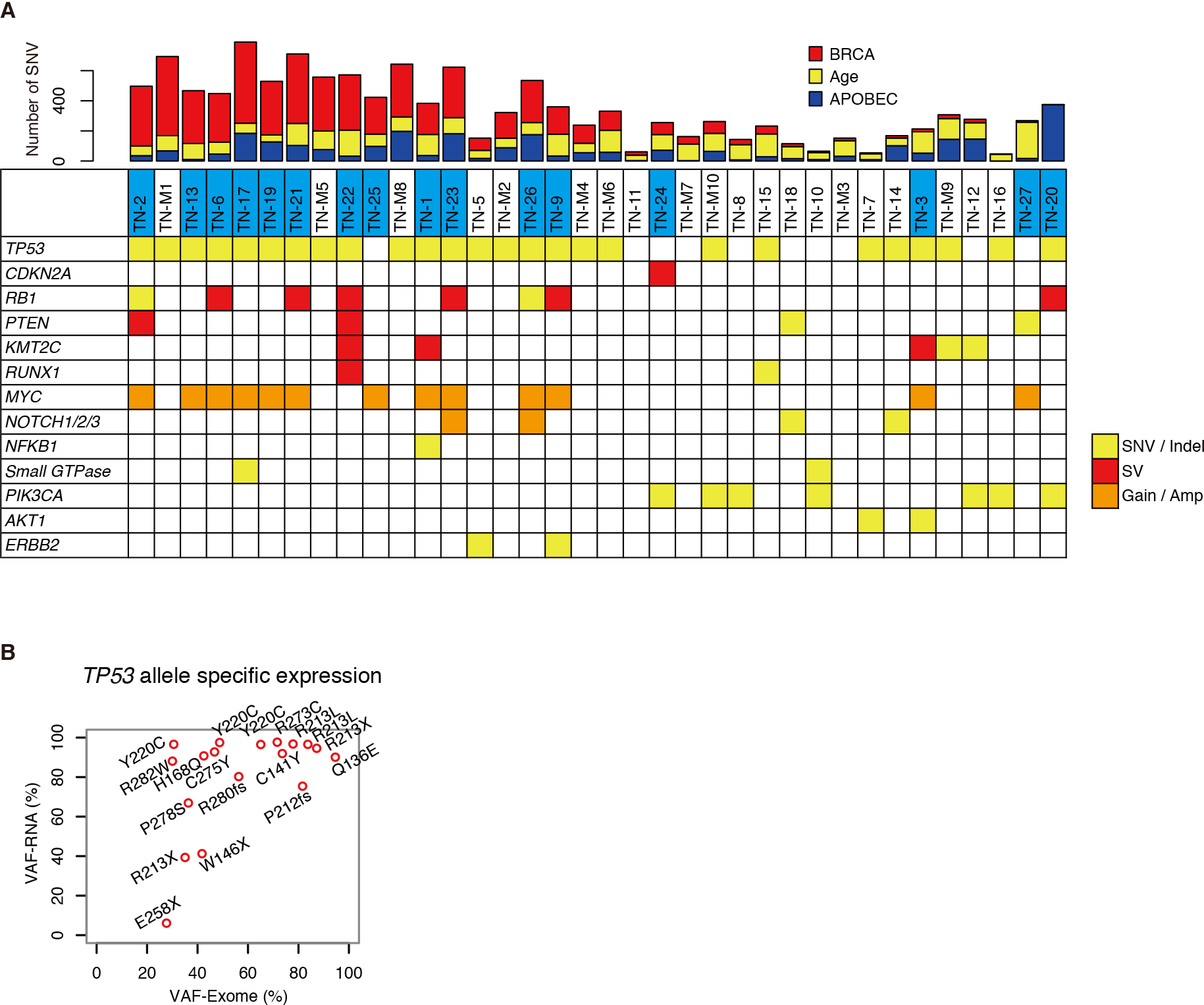

Supplement: S1 Fig — (A) Numbers of single nucleotide variations (SNVs) identified by whole exome sequencing (WES). Data are arranged in descending order of the BRCA signature SNV ratio. IDs of samples subjected to whole genome sequencing (WGS) are indicated by a cyan shadow. Mutations of well-known tumor suppressors and driver oncogenes are shown below. Mutations identified by WGS are also included: yellow, SNV and indel; red, structural variation (SV); orange, copy number gain or amplification. (B) Variant allele frequencies (VAFs) of TP53 mutations among RNA-seq reads plotted against VAFs among WES reads. (TIF) [file pgen.1006853.s001.tif]

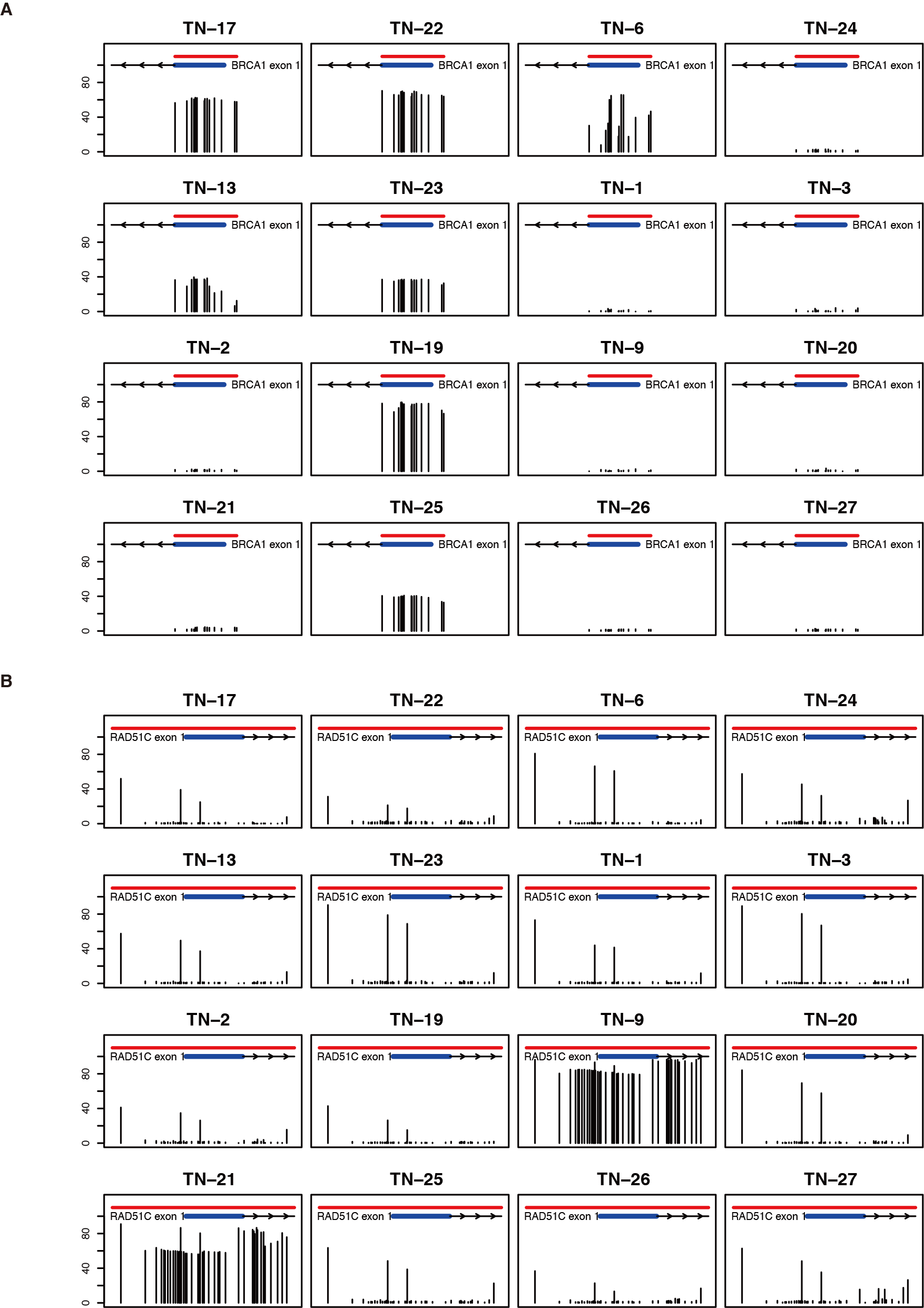

Supplement: S2 Fig — The methylation status of CpG dinucleotides in each patient, as assessed by bisulfite sequencing. The proportion of methylated alleles at each cytosine residue is presented as a vertical line. Red bars indicate analyzed regions. Blue lines indicate exon 1 of BRCA1 (A) and RAD51C (B). (TIF) [file pgen.1006853.s002.tif]

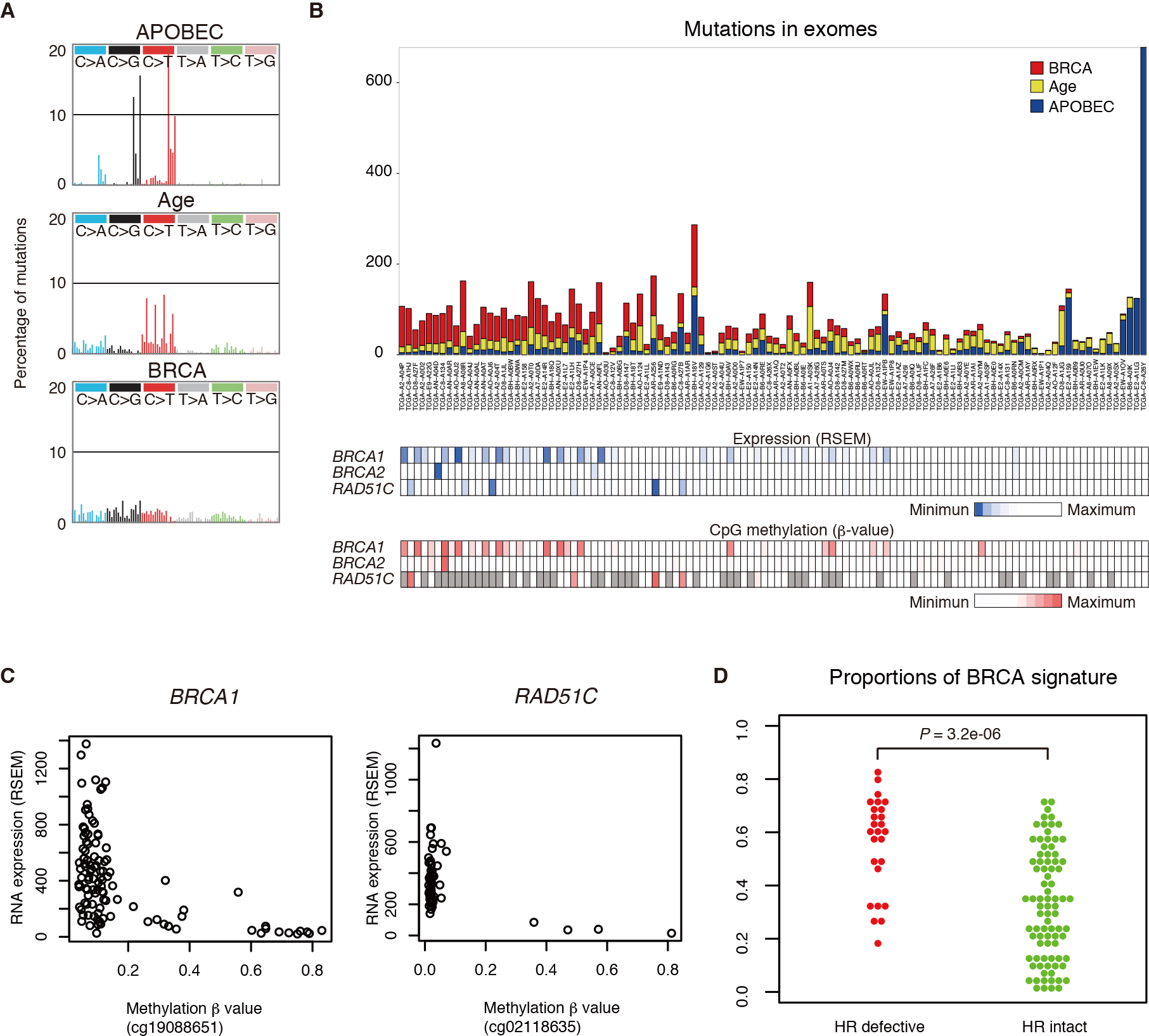

Supplement: S3 Fig — (A) Three trinucleotide mutational signatures identified by analysis of SNVs. (B) Numbers of SNVs in association with mRNA expression and promoter methylation of BRCA1 and RAD51C. Data are arranged in descending order of BRCA signature SNV ratios. Expression status is color-scaled: blue, low. Methylation status is color-scaled: red, high; gray, no data. Data from probes cg19088651 (BRCA1), cg27253386 (BRCA2), and cg02118635 (RAD51C) are shown. (C) mRNA expression of BRCA1 and RAD51C plotted against methylation levels. The threshold for probes cg19088651 and cg02118635 was set to 0.2. (D) Proportions of BRCA signatures. It was assumed that the homologous recombination (HR) pathway was defective when the BRCA1 (cg19088651) or RAD51C (cg02118635) methylation β value was more than 0.2 or BRCA1 harbored a deleterious somatic mutation. Information about germline mutations was not available. (TIF) [file pgen.1006853.s003.tif]

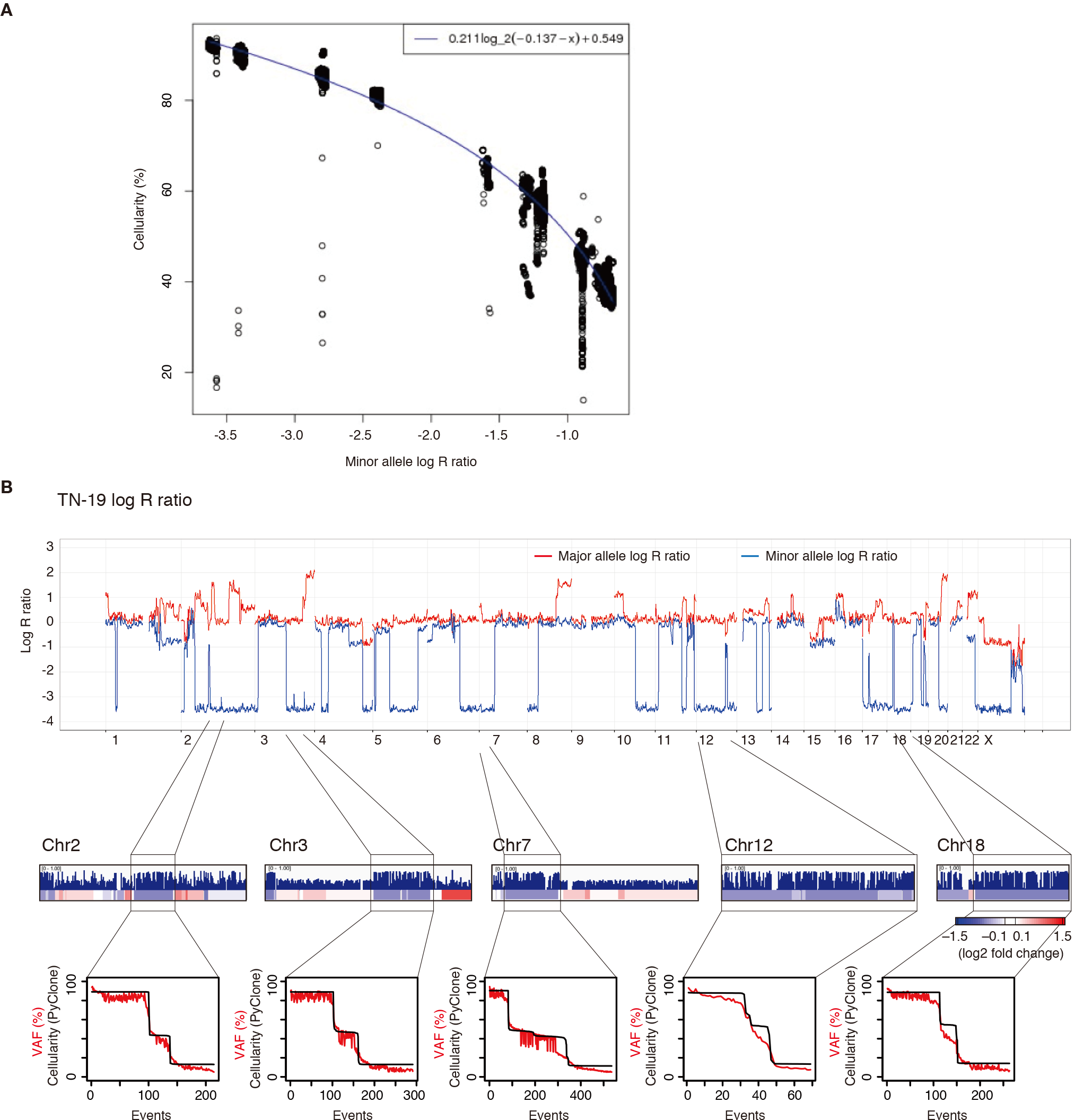

Supplement: S4 Fig — (A) Tumor cellularity deduced from the minor allele proportion plotted against minor allele Log R ratios at all regions where the copy number (CN) of the major allele was one and the CN of the minor allele was zero. (B) Red and blue lines indicate the log R ratios of major and minor alleles, respectively (upper panel). Single nucleotide variations (SNVs; blue vertical lines) are shown along with the CN status (middle panels). The height of each line represents the variant allele frequency (VAF). Clonal analysis of selected regions where one allele was lost is shown (lower panels): red, observed VAFs; black, cellularity predicted using PyClone. (TIF) [file pgen.1006853.s004.tif]

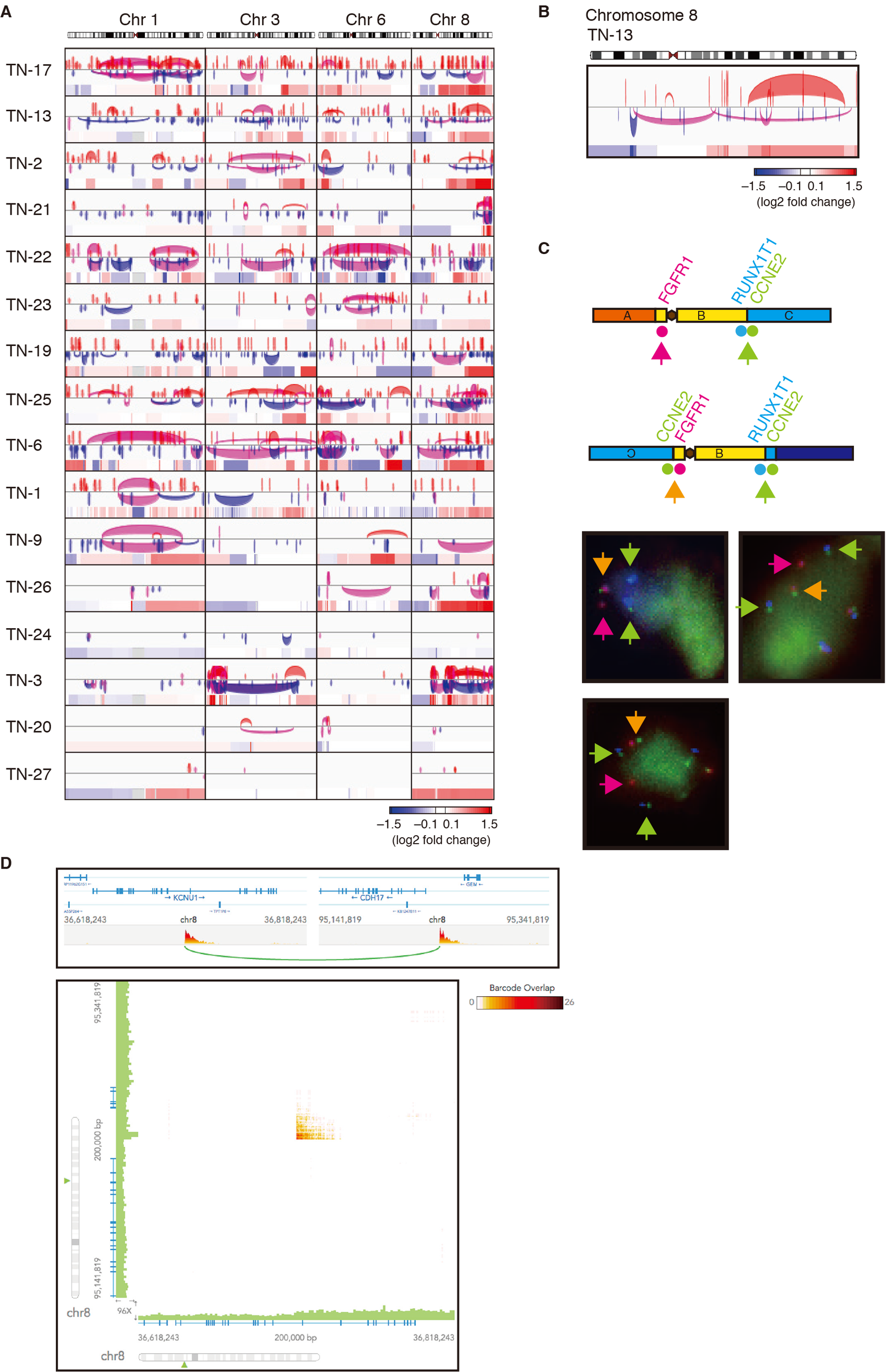

Supplement: S5 Fig — (A) Structural variants (SVs) along with the copy number (CN) status. Each pair of break points constituting an SV is connected by a color-coded arch: red, tandem duplication; magenta, inverted rearrangement; blue, deletion. Note that arches for small SVs appear to be vertical lines owing to limited resolution. The CN status is color-scaled: red, gain; blue, loss. Chromosomes 1, 3, 6, and 8 are shown as representative examples. (B) The status of chromosome 8 in TN-13 as a representative example of a high resolution image. (C) Three-color fluorescence in situ hybridization (FISH) analysis of the TN-19 specimen. FGFR1, RUNX1T1, and CCNE2 loci were detected with Texas red-, Cy5-, and FITC-labeled probes, respectively. Representative high power fields are shown. Presumed structures of chromosome 8 in TN-19 are shown schematically in the upper panel. Brown hexagons indicate centromeres. Solid circles indicate the probes: magenta, FGFR1; cyan, RUNX1T1; green, CCNE2. Colored arrowheads indicate combinations of adjacent probes. (D) Validation of the inverted rearrangement in TN-19 by linked-read sequencing. Heat map of overlapping barcodes plotted for inverted rearrangement on chromosome 8 in TN-19 is shown (bottom panel). Linearized view of the barcode overlap matrix is also shown (upper panel). (TIF) [file pgen.1006853.s005.tif]

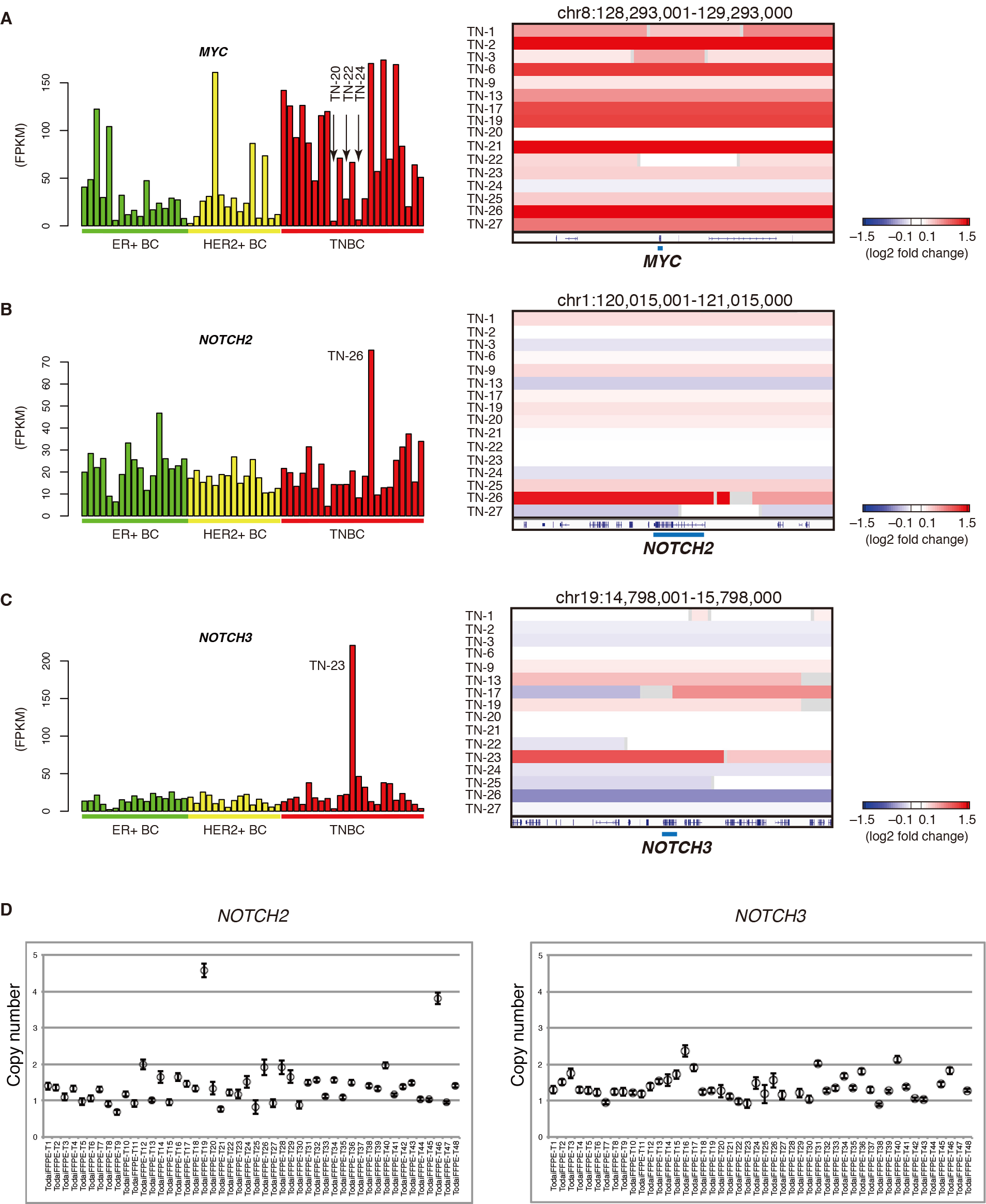

Supplement: S6 Fig — (A–C) mRNA expression of MYC (A), NOTCH2 (B), and NOTCH3 (C) in ER+ breast cancer (BC) (green), HER2+ BC (yellow), and TNBC (red) are shown (left panel) along with the copy number (CN) status of TNBC samples analyzed by whole genome sequencing (right panel). Horizontal blue lines indicate gene loci. The CN status is color-scaled: red, gain; blue, loss. (D) CN values of NOTCH2 and NOTCH3 estimated by droplet digital PCR in 48 FFPE samples. (TIF) [file pgen.1006853.s006.tif]

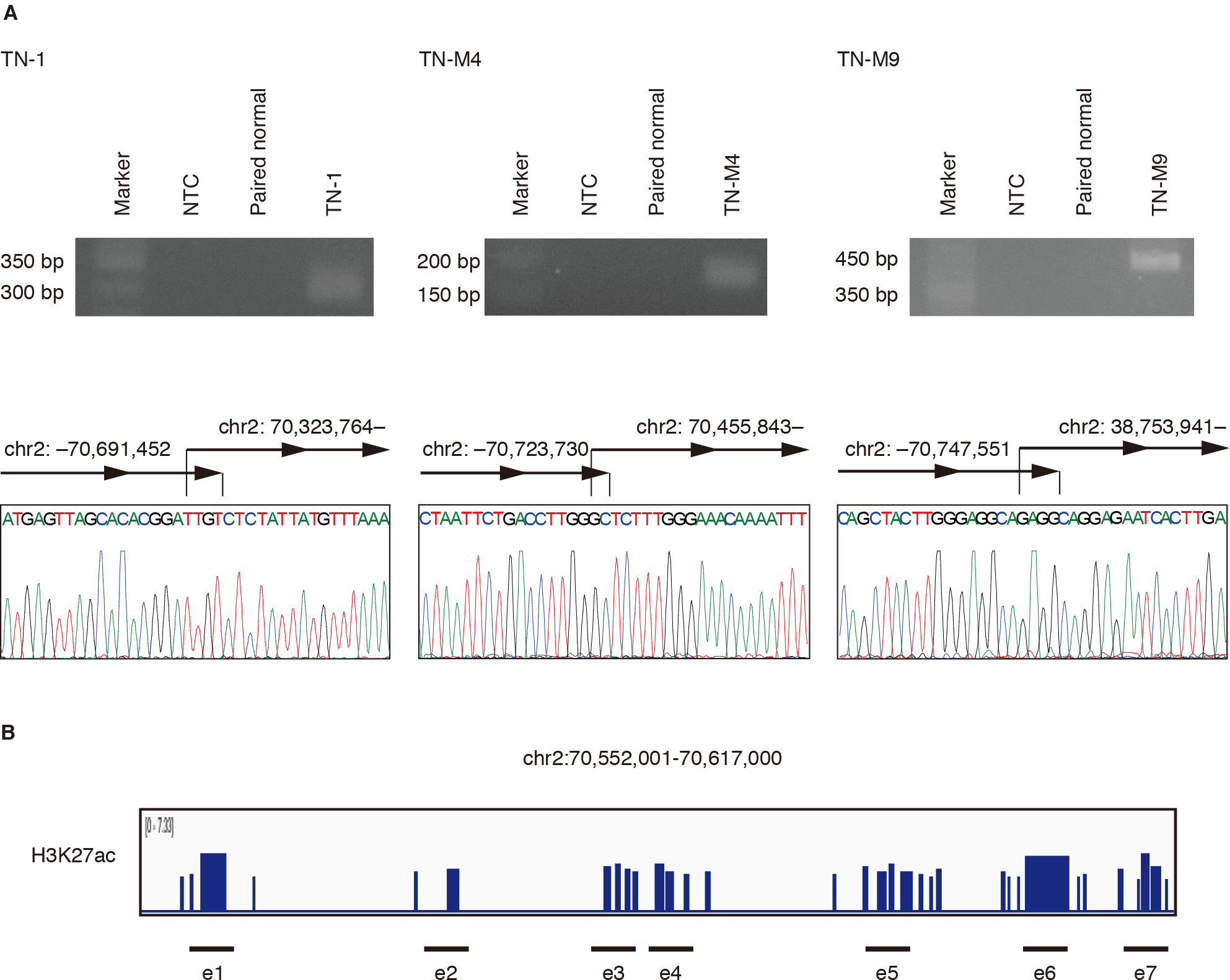

Supplement: S7 Fig — (A) Breakpoints associated with tandem duplications near the TGFA locus were amplified by PCR of genomic DNA from patients TN-1, TN-M4, and TN-M9, followed by Sanger sequencing analysis. (B) Acetylation of the lysine residue at position 27 of histone H3 (H3K27ac) in BICR6 cells detected by ChIP-seq. Putative TGFA regulatory regions are indicated (e1–e7). (TIF) [file pgen.1006853.s007.tif]

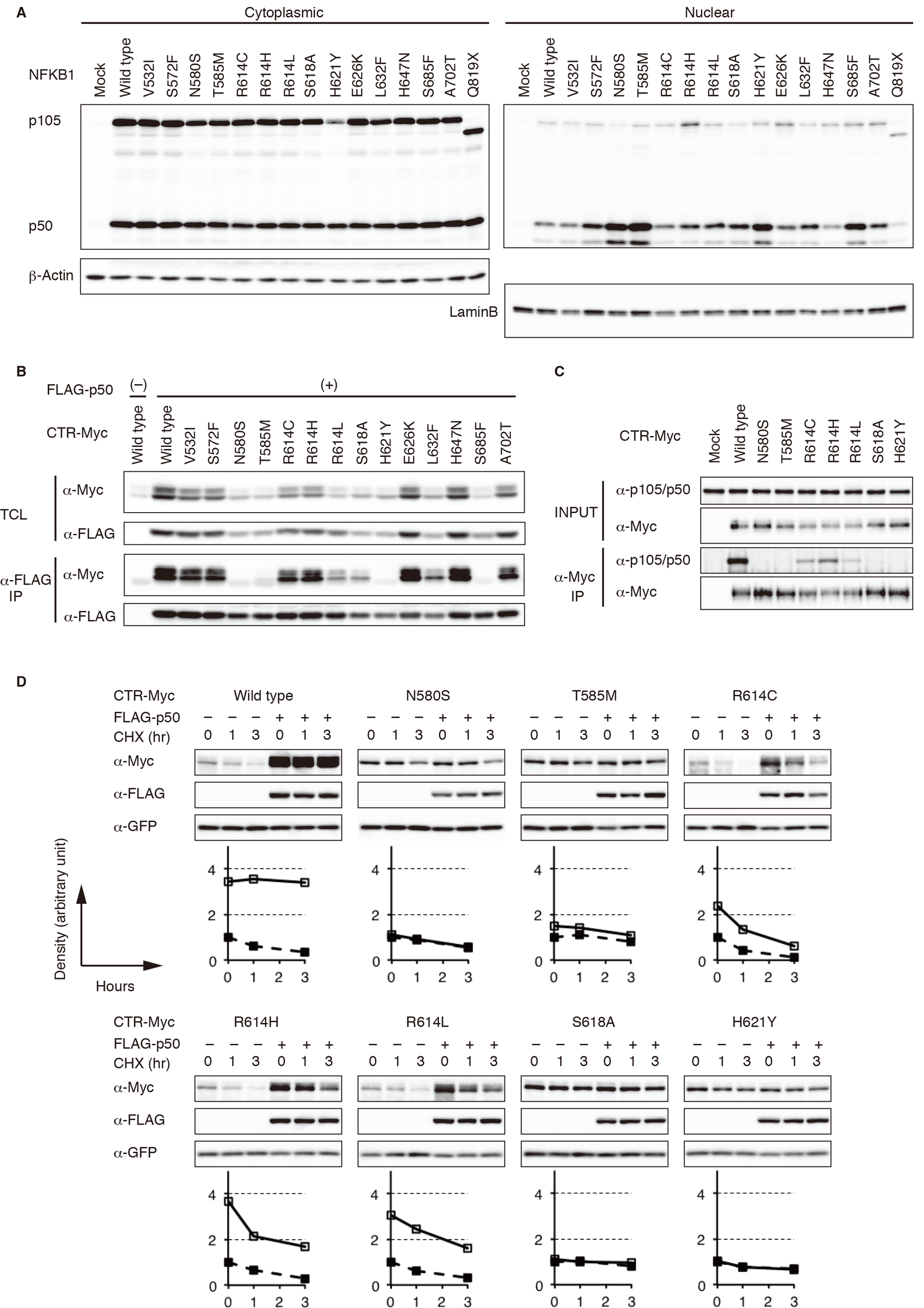

Supplement: S8 Fig — (A) Mouse 3T3 cells were infected with an empty retrovirus (Mock) or recombinant retrovirus encoding either wild-type or mutant forms of NFKB1. Cytoplasmic (left panel) and nuclear (right) fractions of these cells were prepared and subjected to immunoblot analysis with antibodies against p105/p50, lamin B, or β-actin, as indicated. (B) HEK293T cells were transfected with a vector encoding either wild-type or mutant forms of the C-terminal region of NFKB1 tagged with the Myc peptide (CTR-Myc) along with an empty expression vector (–) or a vector encoding FLAG-tagged p50 (+). Total cell lysate (TCL) extracted from transfected cells was subjected to immunoprecipitation (IP) with an anti-FLAG antibody. TCL and immunoprecipitated fractions were analyzed using antibodies against Myc peptide and FLAG peptide. (C) HEK293T cells were transfected with a mock vector or a vector encoding either wild-type or mutant forms of CTR-Myc. TCL was subjected to immunoprecipitation with an anti-Myc antibody and then subjected to intensive washing. Purified wild-type and mutant forms of CTR-Myc were mixed with recombinant p50 (INPUT). CTR-Myc was pulled down and analyzed by immunoblotting. (D) HEK293T cells were transfected with a vector encoding either wild-type or mutant forms of CTR-Myc along with an empty expression vector (–) or a vector encoding FLAG-tagged p50 (+). Cells were treated with 10 μg/ml cycloheximide (CHX) to inhibit protein synthesis for the indicated duration. TCL was analyzed by immunoblotting to assess the stability of proteins. The amount of CTR-Myc measured by densitometry is shown below. GFP was used as a loading control from which the density of CTR-Myc was calculated. Compensated values of CTR-Myc in the absence of p50 at 0 h are set to 1: CTR-Myc with p50, white squares; CTR-Myc without p50, black squares. (TIF) [file pgen.1006853.s008.tif]

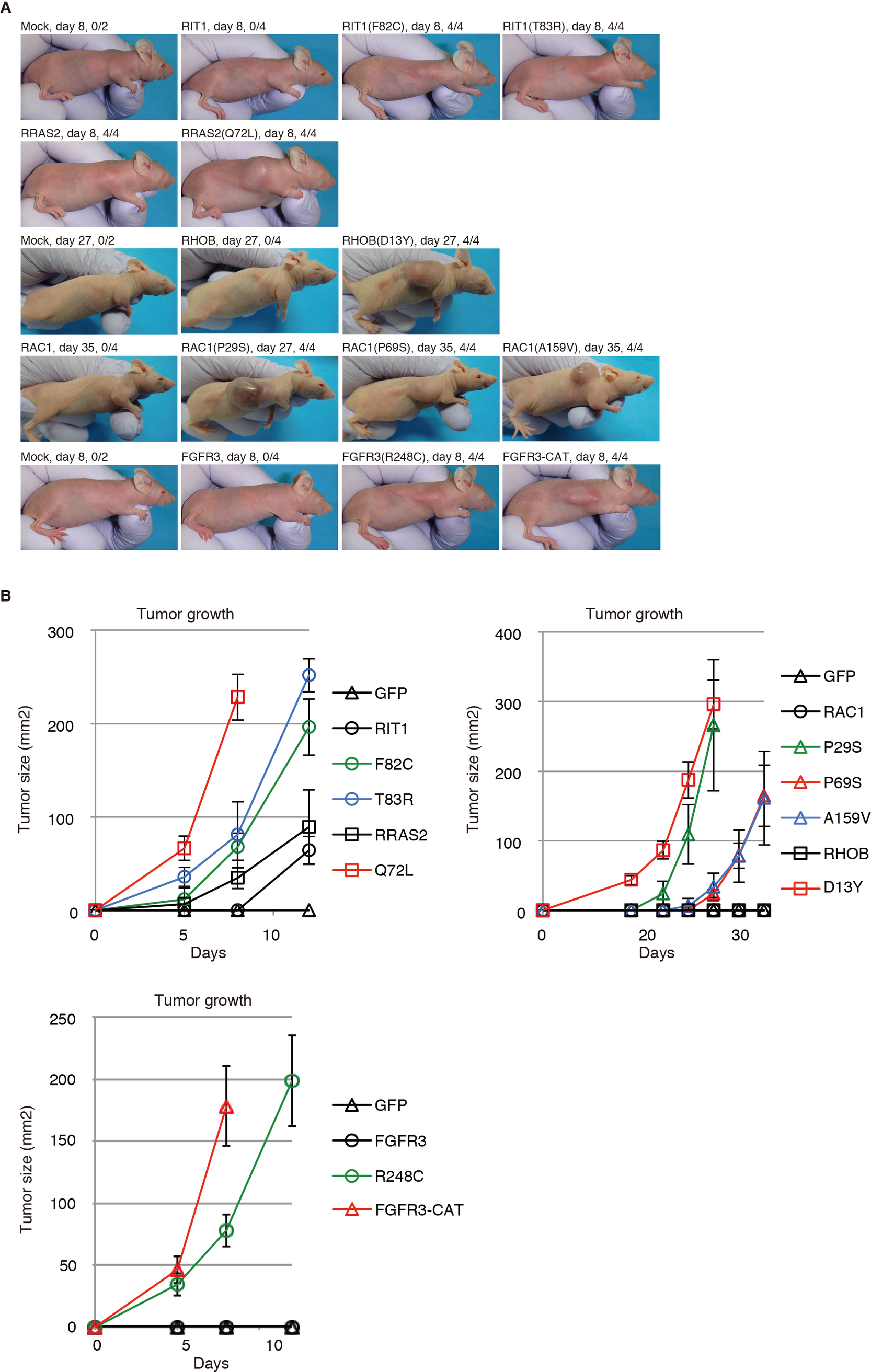

Supplement: S9 Fig — 3T3 cells expressing wild-type or mutant forms of the indicated proteins were injected subcutaneously into the shoulders of nude mice. (A) Representative images of tumors at the indicated times. The numbers of generated tumors (number of generated tumors/number of injection sites) are indicated. (B) Tumor sizes [(length × width)] at the indicated times. Data are the means ± standard deviation. (TIF) [file pgen.1006853.s009.tif]
